# Alterations in Gut Microbiota Correlate With Hematological Injuries Induced by Radiation in Beagles

**DOI:** 10.1155/ijm/3096783

**Published:** 2024-12-03

**Authors:** Zongyu Huang, Likun Wang, Jianghui Tong, Yong Zhao, Hui Ling, Yazhou Zhou, Yafang Tan, Xiaohui Xiong, Yefeng Qiu, Yujing Bi, Zhiyuan Pan, Ruifu Yang

**Affiliations:** ^1^State Key Laboratory of Pathogen and Biosecurity, Academy of Military Medical Sciences, Fengtai, Beijing 100071, China; ^2^College of Food Science and Light Industry, Nanjing Tech University, Nanjing 210009, Jiangsu, China; ^3^Department of Microbiology, School of Basic Medical Sciences, Anhui Medical University, Hefei 230000, Anhui, China; ^4^Laboratory Animal Center, Academy of Military Medical Sciences, Fengtai, Beijing 100071, China; ^5^Department of Research and Development, Grand Life Sciences Group Ltd., China Grand Enterprises Inc., Chaoyang, Beijing 100101, China

**Keywords:** acute radiation syndrome, beagles, gut microbiota, hematological injuries, KEGG pathways, metagenomics

## Abstract

Dynamics of gut microbiota and their associations with the corresponding hematological injuries postradiation remain to be elucidated. Using single whole-body exposure to ^60^Co-*γ* ray radiation at the sublethal dose of 2.5 Gy, we developed a beagle model of acute radiation syndrome (ARS) and then monitored the longitudinal changes of gut microbiome and hematology for 45 days. We found that the absolute counts of circulating lymphocytes, neutrophils, and platelets were sharply declined postradiation, accompanied by a largely shifted composition of gut microbiome that manifested as a significantly increased ratio of *Firmicutes* to *Bacteroidetes*. In irradiated beagles, alterations in hematological parameters reached a nadir on day 14, sustaining for 1 week, which were gradually returned to the normal levels thereafter. However, no structural recovery of gut microbiota was observed throughout the study. Fecal metagenomics revealed that irradiation increased the relative abundances of genus *Streptococcus*, species *Lactobacillus animalis* and *Lactobacillus murinus*, but decreased those of genera *Prevotella* and *Bacteroides*. Metagenomic functions prediction demonstrated that 26 altered KEGG pathways were significantly enriched on Day 14 and 35 postradiation. Furthermore, a total of 43 bacterial species were found to correlate well with hematological parameters by Spearman's analysis. Our results provide an insight into the longitudinal changes in intestinal microbiota at different clinical stages during ARS in canine. Several key microbes those tightly associated with the hematological alterations may serve as biomarkers to discriminate the different phases of host with ARS.

## 1. Introduction

Acute radiation syndrome (ARS) severely endangers health and life: it commonly happens after a whole-body or a partial-body exposure to the high-dose irradiation [1], such as radiation therapy, nuclear accidents, and even radiological terrorist attack [2, 3]. In clinic, ARS commonly clusters with multiorgan damages, involving hematopoietic, gastrointestinal, cardiovascular, and central nervous systems, which poses a considerable medical challenge to physicians [4, 5]. Of which, the injuries of blood cells have been identified as the most sensitive pathophysiological indicators for the radiation-induced effects, characterizing by the decreased counts of lymphocytes, granulocytes, and platelets (PLTs) [6].

Intriguingly, a growing body of evidence conforms that there is an essential link between the gut dysbiosis and the radiation-induced injury [7, 8]. It has been reported that gut microbes in humans are significantly altered after ionizing radiation from a medical exposure [9, 10], which may contribute to the gastrointestinal toxicity after radiation [11, 12]. Previous laboratory studies also demonstrated that radiation exposure durations with divergent dosages apparently bear on gut microbiome [13, 14]. Liu reported that using low-dose ionizing radiation with total level of 0.5 Gy (0.5 Gy × 1 dose, 0.1 Gy/dose × 5 doses twice per week, or 0.05 Gy/dose × 10 doses once per week) significantly affected the composition of the gut microbiota and metabolomes in mice at least for 35 days [13]. Another report showed that intestinal tissue damage and altered gut microbiota were both observed after irradiation for 10 days with a single dose of 6 Gy of gamma rays in mice, and supplementation with *Lactobacillus acidophilus* enhanced intestinal epithelial function by improving stem cell function and cell differentiation [14]. Multiomics analyses of radiation survivors in mice suggested that *Lachnospiraceae* and *Enterococcaceae* could serve as the beneficial microbes to afford protection against radiation [8]. Nevertheless, there is no relevant research on the relationship between hematological indicators and gut microbiota under radiation exposure.

In this regard, several experimental animal models have been developed to screen and explore the agents against radiation-induced damage [15]. However, currently available evidence was mainly originated from the rodent models, and more robust data based on the model of a large outbred species is still urgently needed. Beagles have been considered as the suitable model for the preclinical studies in radiation, since the radiation-induced hematopoietic responses in canines and humans are quite similar [16, 17]. Therefore, developing an ARS model in beagles to investigate the dynamic of gut microbial community and its relationship with hematological injuries is warranted.

In current study, we firstly developed an ARS model in beagles with whole-body single radiation exposure to ^60^Co-*γ* ray. Then, we determined the dynamic of gut microbiome for 45 days by metagenomic sequencing and explored its associations with hematology by correlation analysis.

## 2. Materials and Methods

### 2.1. Animals

Ten male beagle dogs, one-year-old, weighing 9∼10 kg, were obtained from Xi'an Dilepu Biology Resources Development Co. Ltd (Xi'an, China). After calculation, the sample size should be greater than or equal to 5. Thus, we selected ten dogs. Dogs were individually housed in cage racks (74 cm long, 47.5 cm wide, and 214 cm high) with clean bedding at the Experimental Animal Center under a 12-h light/dark cycle at a temperature of 25°C and relatively humidity of 50%, with free access to water. We feed the dog twice a day based on their food intake. Considering the effects of diet on gut microbial community, the drinking water was filtered by the Sterile Experimental Animal Drinking Machine (LINGYUNBOJI Technology Co., Ltd., Beijing), and the dry standard feed for adult canine was sterilized by high temperature at 121°C for 30 min. All animal procedures were performed in accordance with the Declaration of the National Institutes of Health Guide and Use of Laboratory Animals and approved by the animal care and use committee of Beijing Institute of Microbiology and Epidemiology (No. IACUC-DWZX-2018-002).

### 2.2. Study Design and Radiation

After an acclimation period for two weeks, beagles were first fasted for 12 h and then transferred to individual cubes, receiving a single whole-body exposure to ^60^Co-*γ* ray radiation at the Beijing Key Laboratory for Radiobiology (Beijing, China). The dog was in a single cage and no other restrictions on animals during radiation exposure. The total radiation dose was 2.5 Gy with the delivering dose rate at 59.57 cGy/min, and the distance from radiation source to beagles was 450 cm. After irradiation, beagles were sent back to cage racks, followed by an observational period of 45 days. After the experiments, dogs were euthanized by intravenous injection of sodium pentobarbital (dosage 100 mg/Kg).

### 2.3. Sample Collection

Fresh stools and whole blood samples from each animal were harvested at the day of 3, 7, 14, 21, 35, and 45 after exposure to radiation, respectively. To ensure the collection of all preradiation feces, Beagles' fecal samples from the seventh day to the fourth day prior to radiation were collected, which viewed as the baseline (marked as Day 0). Stool samples were collected in sterile tubes from the beagles after they defecate on its own. Then, the stool samples were immediately stored at −80°C for metagenomics sequencing.

We collected blood sample on the third day before radiation. Blood samples were collected in tubes containing ethylenediaminetetraacetic acid (EDTA). During blood collection, the limbs of beagles were fixed with the fixing frame, and blood was taken from the cephalic vein. Then, we used cotton wool to compress the blood to stop bleeding. Hematological indicators, including the levels of red blood cell (RBC), white blood cell, neutrophils, lymphocytes, PLTs, and hematocrit, were determined with an IDEXX ProCyte Dx Analyzer (IDDEXX Laboratories, Inc. USA).

### 2.4. Gut Microbiome Assay

Each stool weighing about 400 mg from beagles before and after radiation was used for assay.

### 2.5. Genomics DNA Extraction

Total microbial genomic DNA was extracted using MagPure Stool DNA KF kit B (Magen, China) according to the manufacturer's instructions. The quality of bacterial genomic DNA was checked by 1% agarose gel electrophoresis and quantified with a Qubit Fluorometer by using Qubit dsDNA BR Assay kit (Invitrogen, USA).

### 2.6. Library Construction

After extraction, 1 μg genomic DNA from each sample was randomly fragmented by Covaris E220 to produce DNA fragments from 200 to 400 bp, followed by purification with the AxyPrep Mag PCR Clean-Up Kit (Axygen, USA). Then, fragments were end-repaired by the End Repair Mix (TruSeq DNA Sample Prep Kit, Illumina, Inc.) and were purified using Agencourt AMPure XP beads (Beckman Coulter, USA). The repaired DNAs were combined with A-Tailing Mix, and then, the Illumina adapters were ligated to the Adenylate 3′Ends DNA and followed by product purification. The products were selected based on the insert size. Several rounds of PCR amplification with PCR Primer Cocktail and PCR Master Mix were performed to enrich the Adapter ligated DNA fragments. After purification, the library was qualified by the Agilent 2100 bioanalyzer (Agilent, USA) and ABI StepOnePlus Realtime PCR System. Finally, the qualified DNA libraries were sequenced on illumina Hiseq platform (BGI, Shenzhen, China).

### 2.7. Metagenomic Data Analysis

All the raw data were trimmed by SOAPnuke v.1.5.2 [18]. The trimmed reads were mapped to the host genome using SOAP2 software to identify and remove host originated reads [19]. High-quality reads were de novo assembled using IDBA-UD software [20]. Assembled contigs with length less than 300 bp were discarded in the following analysis. Genes were predicted over contigs by using MetaGeneMarker (2.10) [21]. Redundant genes were removed using CD-HIT with identity cut-off value 95% [22]. Based on the MEGAN LCA algorithm, the taxonomic annotation was assigned [23]. To generate the taxonomic and functional abundance profiles, the reads were aligned to the genes using Botwie2 with the default setting [24]. Based on the abundance profiles, the features (Genera and Phyla) with significantly differential abundances across time points were determined using Wilcoxon's rank sum test [25]. *p* values for multiple testing were corrected using the BH method, and the corrected *p* values < 0.05 were considered as significance [26]. Differentially enriched KEGG pathways were identified according to reporter scores [27]. An absolute value of reporter score greater than 1.65 was used as the detection threshold for significance. The alpha diversity was quantified by the Shannon index using the relative abundance profiles at gene, bacterial genus, and KO level with R package. The beta diversity was calculated based on Bray–Curtis distance [28]. Principal component analysis (PCA) was plotted with R package “ade4.”

### 2.8. Statistical Analysis

The physiological indicators were plotted by GraphPad Prism 8.0 software. Statistics were performed with one-way analysis of variance followed by unpaired two-tailed Student's *t*-test. If the two sets of data conform to normal distribution and homoscedasticity, the paired *t*-test is used. When ⁣^∗^*p* < 0.05, ⁣^∗∗^*p* < 0.01, ⁣^∗∗∗^*p* < 0.001, and ⁣^∗∗∗∗^*p* < 0.0001, the differences were statistically significant. Spearman correlation coefficients (*p* < 0.05) and heatmaps were carried out to disclose the correlation between gut microbiota and hematology by Graph prism (8.0) software.

## 3. Results

### 3.1. Survival Rates and Changes of Hematology in Irradiated Beagles

To precisely present the dynamics of gut microbiome and hematology, we set the total radiation dose to 2.5 Gy, because such sublethal dosage (2.4 Gy) has been reported to induce the ARS over 45 days in beagles, which could entirely simulate the time course of hematology from acute injuries to recovery throughout this observational period [29]. All beagles exhibited a rapid vomit response within hours after irradiation. One dog died on Day 13 and the other died on Day 19 postradiation, with the final survival rate of 80% on Day 45.

**Figure 1:**
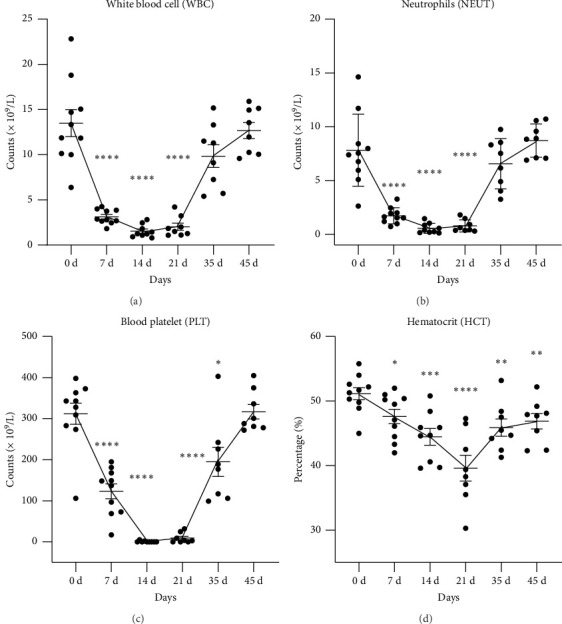
Time course of hematological changes in beagles for 45 days after exposure to an acute, whole body, nonlethal gamma ray at the dose of 2.5 Gy. (a) White blood cell, (b) neutrophils, (c) platelets, and (d) hematocrit. Data were plotted in GraphPad Prism 8.0 software. Statistical significances were performed by one-way analysis of variance followed with paired *t*-test. ⁣^∗^*p* < 0.05; ⁣^∗∗^*p* < 0.01; ⁣^∗∗∗^*p* < 0.001; ⁣^∗∗∗∗^*p* < 0.0001, compared with the level at Day 0, respectively.

Generally, ARS progresses through 4 clinical phases: prodrome, latency, manifest illness, and either recovery or death [30]. The prodromal period is characterized by several gastrointestinal symptoms, whereas the latency period is characterized by partial or complete resolution of symptoms [30]. Our hematological results showed that reductions of the absolute counts of the white blood cells (Figure 1(a)), neutrophils (Figure 1(b)), and PLTs (Figure 1(c)), as well as the hematocrit (Figure 1(d)), were observed from a few days postradiation onward and further reached a nadir on Day 14. In the third phase of ARS (the manifest illness period), the counts of blood cells and PLTs remained almost disappeared from Day 14–21, which finally recovered to the normal levels on Day 45 (Figures 1(a), 1(b), 1(c)). Corresponding statistical values of above hematological data were shown in Table S1. Such longitudinal alterations of hematology in beagles are in accord with the special change pattern of the blood cells in humans after a whole-body exposure to ionizing radiation [6].

### 3.2. Characteristic Alterations in Gut Microbiota in Canine After Radiation

Metagenomics sequencing was applied to monitor the instant variations of intestinal microbiome in canines. Sampling time points were selected matching to those for hematological investigations, but we added a sampling time on Day 3 postradiation.

**Figure 2:**
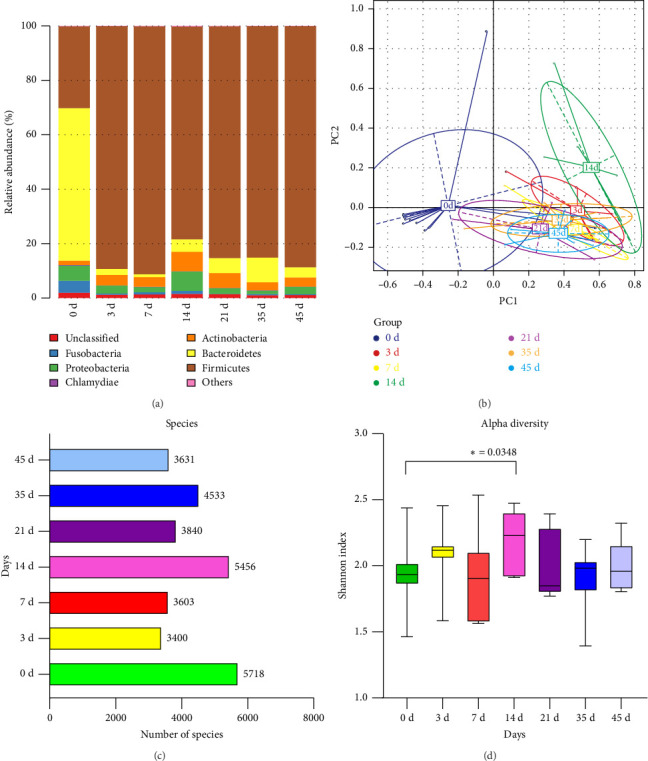
Changes of intestinal microbial community in beagles after an acute, whole body, sublethal gamma ray exposure with 2.5 Gy dosage. (a) Alpha diversity analysis (Shannon index, ⁣^∗^*p* < 0.05, Wilcoxon's rank sum test). (b) Amounts of bacterial species. (c) Beta diversity comparisons based on principal component analysis (PCA), which was plotted with R package “ade4.” (d) Relative abundance of bacteria at phylum level.

We found that alpha diversity (Shannon index) of gut microbiome was significantly increased on Day 14 postradiation compared to that on Day 0, demonstrating a higher evenness and richness of intestinal bacterial species after radiation (Figure 2(a)). However, the amounts of bacterial species in microbial community were immediately decreased within the first 3 days in irradiated beagles, which temporarily reversed on Day 14, but continuously decreased in the following time points (Figure 2(b)). Beta-diversity comparisons by PCA plots of the gut microbiome exerted a max distance between baseline (Day 0) and Day 14 (Figure 2(c)), showing a substantially distinct microbial composition in the latent stage postradiation. Although plots of microbiome on Day 21, 35, and 45 were overlapped in a great degree, they were all dissimilar to that of baseline, respectively (Figure 2(c)). In addition, we also analyzed gut microbiota co-occurrence network which is shown in Figure S1. These results demonstrated that radiation irreversibly altered the structure of gut microbiota in beagles.

For the relative abundance of bacteria at phylum level, we showed that at baseline (Day 0), the canine gut microbiota was dominated by *Bacteroidetes*, followed by the minor abundance of *Firmicutes* (Figure 2(d)). Other relative lower proportions such as *Actinobacteria*, *Proteobacteria*, and *Fusobacteria* were also observed (Figure 2(d)). Notably, the relative abundance of phylum *Bacteroidetes* was sharply declined within the first 3 days after radiation challenge, which maintained at low levels throughout the entire observational period for 45 days (Figure 2(d)). Although the hematological parameters had reverted to the normal levels on day 45 (Figure 1), canine microbial community, especially for the abundance of *Bacteroidetes*, remained no recovery trend.

**Figure 3:**
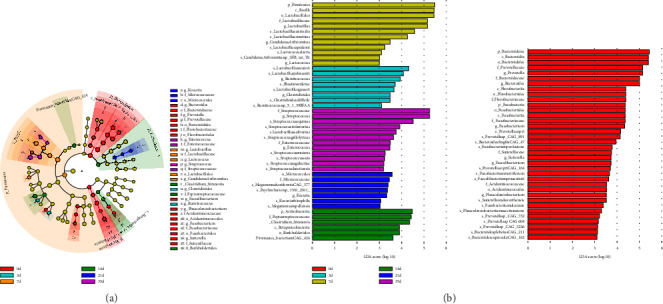
Differentially abundant microbes in irradiated beagles. Linear discriminant analysis (LDA) effect size (LEfSe) analysis was performed on the relative abundance data of the microbial community. (a) Cladogram. (b) LDA score. To uncover the differentially distributed taxa, data from the observed time points were first analyzed by the Kruskal–Wallis test with a significance set to 0.05. Those differentially distributed taxa were then used for LDA model analysis to rank the relative abundance significance. The LDA for significance was set to ±2, and the log(10) transformed score was shown as the effect size.

In irradiated beagles, cladogram generated from the linear discriminant analysis (LDA) effect size (LEfSe) displayed that a total of 33 microbial taxa in phylogenetic distributions from phylum to species were identified as significant differences (Figure 3(a)). According to LDA scores (LDA score ≥ 2, *p* < 0.05), characteristically enriched bacterial species at the divergent clinical phases during ARS were disclosed (Figure 3(b)). At the genus level, higher abundance of *Prevotella, Bacteroides, Fusobacterium, Sutterella,* and *Faecalibacterium* was identified at baseline (Day 0). In the prodromal phase postradiation, relatively higher enrichment of *Clostridioides* and *Ruminococcus* was seen on Day 3. During the latency period, higher abundance of genera *Lactobacillus, Lactococcus*, and *Candidatus Arthromitus* on Day 7 were observed. On day 14 higher abundance were *Enterococcus* and *Streptococcus* at genus level. In the manifest illness phase, the abundance of genus *Kocuria* was apparently raised on Day 21, while the higher abundance of genera *Enterococcus* and *Streptococcus* was occurred at the recovery period (Figure 3(b)).

**Figure 4:**
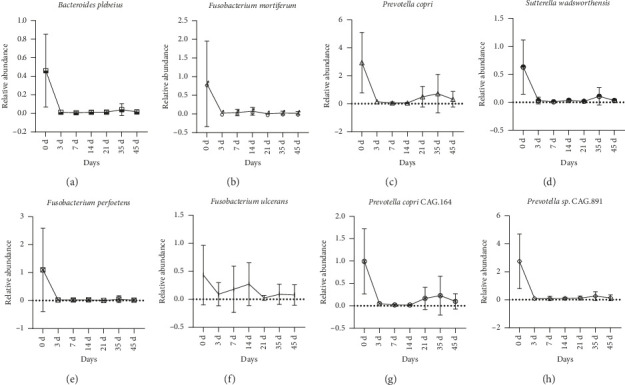
Time course of gut microbes which are highly sensitive to gamma ray radiation in beagles. The relative abundance of each bacterial species was determined by metagenomic sequencing. Data were expressed as mean ± SD. Figures were generated by GraphPad Prism 8.0 software. (a) *Bacteroides plebeius*. (b) *Fusobacterium mortiferum.* (c) *Prevotella copri*. (d) *Sutterella wadsworthensis*. (e) *Fusobacterium perfoetens*. (f) *Fusobacterium ulcerans*. (g) *Prevotella copri CAG.164*. (h) *Prevotella sp. CAG.891*.

At species level, time course of the significantly altered bacteria based on relative abundance analysis (Top 30) is shown in Figure S2. Notably, eight bacterial species of them were found to be highly sensitive to gamma ray exposure (Figure 4), since they almost disappeared without restorative growth after radiation. They were *Bacteroides plebeius* (Figure 4(a)), *Fusobacterium mortiferum* (Figure 4(b)), *Prevotella copri* (Figure 4(c)), *Sutterella wadsworthensis* (Figure 4(d)), *Fusobacterium perfoetens* (Figure 4(e)), *Fusobacterium ulcerans* (Figure 4(f)), *Prevotella copri* CAG.164 (Figure 4(g)), and *Prevotella* sp. CAG.891 (Figure 4(h)).

### 3.3. The Altered KEGG Pathways of Microbiota Metagenomic Function Prediction in Irradiated Beagles

**Figure 5:**
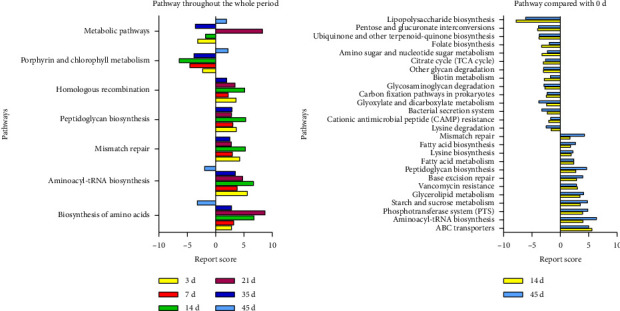
The metagenomic function prediction of the altered pathway in irradiated beagles based on the KEGG database. (a) Comparing with the self-controlled baseline (Day 0), the altered pathways during the entirely observational period were found in irradiated beagles. (b) Comparing to the baseline, the significantly altered pathways were observed postradiation for 14 and 45 days, respectively.

To explore the potential functions of microbial metagenomic, we applied the Kyoto Encyclopedia of Genes and Genomes (KEGG) Orthologys (KOs) database to predict the pathways by which the gut microbiota response to radiation. According to the annotation information of KOs with significantly changed relative abundance of microbes, we found that, compared to baseline (Day 0), pathways of homologous recombination, peptidoglycan biosynthesis, mismatch repair, aminoacyl–tRNA biosynthesis, and biosynthesis of amino acids were upregulated in majority of time points in irradiated beagles, except for those pathways of aminoacyl–tRNA biosynthesis and biosynthesis of amino acids were downregulated on Day 45 (Figure 5(a)). By contrast, pathway of porphyrin and chlorophyll metabolism was upregulated on Day 45 but downregulated in the other time points in irradiated beagles. Regarding the metabolic pathways, they were upregulated on Day 21 and 45 whereas downregulated on Day 3, 14, and 35 (Figure 5(a)).

By comparing with the self-controlled baseline, we further focused on the significantly changed KEGG pathways at level two between pre- and postradiation. Time points of Day 14 and Day 45 were selected because they represented for the phase of manifest illness phase and the recovery phase during ARS, respectively. Compared to baseline (Day 0), a total of 26 altered KEGG pathways were predicted both on Day 14 and Day 45, with 12 upregulated and 14 downregulated (Figure 5(b)). After radiation, pathways of mismatch repair, fatty acid biosynthesis, lysine biosynthesis, fatty acid metabolism, peptidoglycan biosynthesis, base excision repair, vancomycin resistance, glycerolipid metabolism, starch and sucrose metabolism, phosphotransferase system, aminoacyl-tRNA biosynthesis, and ABC transporters were all upregulated while pathways of lipopolysaccharide biosynthesis, pentose and glucuronate interconversions, ubiquinone and other terpenoid-quinone biosynthesis, folate biosynthesis, amino sugar and nucleotide sugar metabolism, citrate cycle, other glycan degradation, biotin metabolism, glycosaminoglycan degradation, carbon fixation pathways in prokaryotes, glyoxylate and dicarboxylate metabolism, bacterial secretion system, cationic antimicrobial peptide resistance, and lysine degradation were downregulated (Figure 5(b)).

### 3.4. Correlations Between Hematology and Gut Microbiome

**Figure 6:**
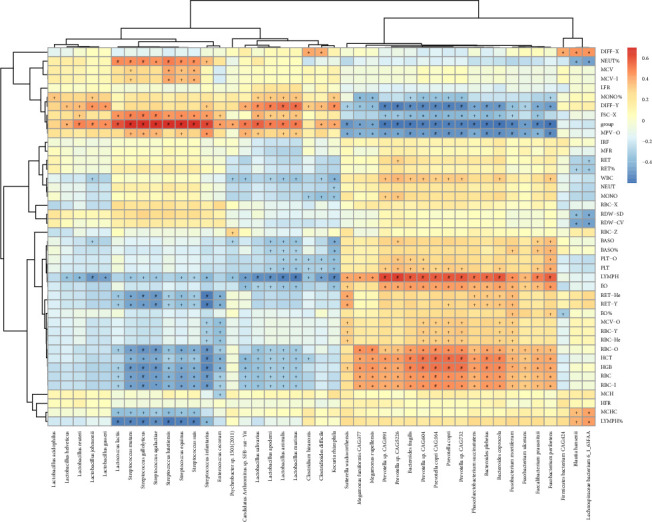
Spearman correlation coefficients between hematology and gut microbiota in irradiated beagles. The *X* axis shows the 43 bacterial species, while the *y* axis shows the 41 hematological parameters and 1 variable (group). Each column represents a different bacterial species identified by metagenomics sequencing in stool samples in beagles. Red indicates positive correlation, whereas blue indicates negative correlation. The darker values are associated with the stronger correlations. ^+^*p* < 0.05; ⁣^∗^*p* < 0.01; ^#^*p* < 0.001.

To explore the potential interactions between the hematological injuries and gut microbes in irradiated beagles, a total of 43 significantly altered bacterial species during ARS together with 41 hematological indicators were selected to calculate the Spearman correlation coefficients, which was visualized in a clustered heat map (Figure 6). Notably, the absolute counts of RBC, PLTs, lymphocytes (LYMPH), eosinophils (EO), basophils (BASO), as well as the levels of hemoglobin (HGB) and hematocrit (HCT) positively correlated with most of the altered bacterial species belonging to the genera *Prevotella*, *Bacteroides*, and *Fusobacterium*. However, these hematological parameters negatively correlated with most of the altered bacterial species belonging to the genera *Lactobacillus* and *Streptococcus*. In contrast, differential white blood cells classification (DIFF-Y), mean platelet volume (MPV) and monocyte proportions (MONO%) positively correlated with most of the genera Lactobacillus and *Streptococcus*, but negatively correlated with the genera *Prevotella*, *Bacteroides*, and *Fusobacterium*.

## 4. Discussion

It has been well recognized that gut microbiota plays a crucial role in health and disease [31]. Interestingly, accumulating evidence suggests that irradiation heavily alters the gut microbes [32], which may conversely affect the recovery of radiation-induced tissue damage [11]. In this field, most investigations examining the effects of radiation on gut microbiota were conducted in cancer patients with radiotherapy [33, 34]. There is still lack of research regarding gut microbiota dynamic after a radiological or nuclear mass casualty incident under the simulating condition of public health emergency. Therefore, in the current study, we firstly developed a canine model of ARS through single whole-body irradiation with ^60^Co-*γ* ray, which entirely mimics the clinical process from hematological injury to recovery in survivors after acute radiation exposure.

In general, higher exposed dosage causes more severe radiation-induced injury [35]. However, many variable factors, such as chronic or acute exposure, dose delivering rate, and experimental subjects, will result in different outcomes in radiation studies, even with the equal exposed dosage. Considering the fact that acute whole-body gamma ray irradiation caused 1 death in 20 dogs at the single dose of 2.5 Gy [15], we therefore chose such sublethal dosage to radiate animals and finally achieved the survival of 80% in irradiated beagles. As expected, the changes of hematology in the surviving dogs were highly consistent with the clinical observations in humans with ARS [36, 37], showing a fashion that irradiation sharply declined the absolute counts of circulating lymphocytes, neutrophils, and PLTs, which gradually reverted to the normal levels thereafter.

The composition of gut microbiota in humans and animals is indeed affected by irradiation, but the details of intestinal bacterial species during the ARS are still poorly characterized. In the present study, one of the most important features regarding the altered gut microbial community postradiation was the dramatically decreased abundance of the phylum *Bacteroidetes* without obvious recovery, even though hematological parameters were returned to the normal levels at the corresponding time point. Currently, the impacts of irradiation on the abundance of *Bacteroidetes* are still controversial. In the large animal models, irradiation profoundly disrupted gut microbiota profiles, leading to an opposite change for the phylum *Bacteroidetes*, with decreased abundance in minipigs but increased abundance in rhesus macaques [38, 39]. In rodents, using 16S rDNA amplicon sequencing, total-body radiation with ^60^Co significantly decreased the *Firmicutes*/*Bacteroidetes* ratio, which was mainly owing to the increased abundance of phylum *Bacteroidetes* postradiation for 14 days [40]. In contrast, a time dependently decreased abundance of phylum *Bacteroidetes* was recently described in male BALB/c mice after low-dose ^60^Co radiation, accompanied by the decreased number of species as well as an increased alpha diversity [13]. This finding was in line with our results that radiation led to a higher Shannon index on Day 14 but decreased amounts of bacterial species in most of time points postradiation. In patients undergoing pelvic radiotherapy, a lower abundance of phylum *Bacteroidetes* was also identified in fecal samples than that in control group [41]. These conflicting results might be attributed to differences in the experimental subjects, sequencing method, and operational conditions. As we known, radiosensitivity differs between animal models [42], and the microbiota is also known to vary between species [43]. It has been shown that gut microbiota in healthy mice exhibits a higher ratio of *Firmicutes*/*Bacteroidetes* than that in beagles [44], and several studies demonstrate that comparing to mice, gut microbiota in beagle or pig is more similar to that in humans [45, 46]. Moreover, we noticed that healthy beagles used in another study had a higher abundance of *Firmicutes* than those in this study [47]. Altogether, these results indicate that animal models used for gut microbiota research in radiation should be chosen with caution.

We found that the beta-diversity of gut microbiota in irradiated beagles remained significantly altered after challenge for 45 days. Likewise, a prospective study in patients with gynecologic cancers displayed that the structure and composition of gut microbiomes remained significantly altered during the 12 weeks follow-up period after receiving pelvic radiation [48]. Also, it has been revealed that after radiation challenge for 280 days, bacterial community in irradiated mice was still distinct from that of controls [8]. Collectively, these results support the notion that irradiation elicits a long-lasting and not easily recovered effect on intestinal microbial community.

Our further analysis implied that the abundances of genera *Prevotella*, *Bacteroides*, *Fusobacterium*, *Sutterella*, and *Faecalibacterium* were all significantly decreased throughout the study compared to those before radiation. Currently, the evidence for the radiation on these genera is quite limited, especially for that on genus *Sutterella*. The abundance of genus *Bacteroides* in minipigs and genus *Prevotella* in macaques was shown to be profoundly decreased by irradiation [47]. However, another report argued that genera *Bacteroides* and *Prevotella* were both increased in nonhuman primates (Chinese rhesus macaques, Macaca mulatta) after radiation with high dose for 4 days [39]. In addition, a study investigating the impact of pelvic radiotherapy on gut microbiota of gynecological cancer patients using pyrosequencing of bacterial 16S rRNA fragments showed that *Fusobacteriaceae* abundance increased after radiation [49]. Other reports demonstrated that ionizing radiation exposure would decrease the beneficial bacteria such as *Faecalibacterium* [50, 51], which agreed with our results.

Using metagenomics sequencing, we identified several species that were the key players contributing to the decreased abundances of bacterial genera after radiation challenge (Figure 4). In irradiated beagles, the decreased abundance of genus *Bacteroides* was primarily attributed to the deletion of *Bacteroides plebeius*. The reduction of genus *Fusobacterium* was mainly due to the sharp declines of *Fusobacterium mortiferum*, *Fusobacterium perfoetens*, and *Fusobacterium ulcerans*. The change in genus *Prevotella* originated from the drops of *Prevotella copri*, *Prevotella copri* CAG.164, and *Prevotella sp.* CAG.891. While the decline of genus *Sutterella* was driven by disappearing of *Sutterella wadsworthensis*. To our knowledge, *Bacteroides plebeius* were usually found in human gut microbiota which can produce porphyranase, an enzyme that can break down porphyan [52]. *Sutterella wadsworthensis* was found in both human and dog gut microbiota, and some studies demonstrated that *Sutterella wadsworthensis* can induce IL-8 production in enterocytes [53]. *Prevotella copri* had been proved to be both positive and negative to host healthy while *Fusobacterium mortiferum* can cause infection cases [54, 55]. The other species of *Fusobacterium perfoetens*, *Fusobacterium ulcerans*, *Prevotella copri* CAG.194, and *Prevotella sp.* CAG.891 had few information about their effects on host, which needed to be further investigated.

Another key finding in current study is that characteristic alterations in gut microbes greatly differ in the various phases during ARS. Many studies attempted to explore the intestinal microbiota as novel biomarkers after acute radiation exposure. It has been documented that radiation decreased the relative abundances of genera *Lactobacillus*, *Ruminococcus*, and *Streptococcus* in irradiated mice with a total dose of 4 Gy of ^60^Co-*γ* rays [56]. However, radiation was also displayed to cause significant increase of genus *Lactobacillus* as well as families *Lactobacillaceae* and *Streptococcaceae*, but decrease of several *Clostridiaceae* members, and failed to change the *Peptostreptococcaceae* in male Wistar rats after whole-body irradiation [57]. Similarly, radiation also increased the proportion of *Lactobacillus* in the large intestine in mice [58]. Nevertheless, they did not clarify the characteristics of microbes among different clinical stages during ARS. Here, we delineated the dynamics of these characteristic alterations of bacterial species after radiation, showing that at the latency phase, the predominant change in gut microbiota is the enrichment of genera *Lactobacillus*, *Ruminococcus*, and *clostridioides*. While at the manifest illness phase, the main features in gut microbiota are higher relative abundances of families *Micrococcales* and *Peptostreptococcaceae*. At the recovery phase, the characteristically enriched genera *Streptococcus and Enterococcus* were observed (Table 1). These key microbes varied at different phases may directly produce divergent regulations on the KEGG pathways using metagenomic function prediction based on (KEGG) Orthologys (KOs) database. We found that in irradiated beagles, the upregulated pathways of peptidoglycan biosynthesis, homologous recombination and mismatch repair were observed. Several studies had proved that homologous recombination and mismatch repair were strongly affected by the gamma ray irradiation [59, 60]. With the change of amino acids biosynthesis pathway, several events may occur such as epigenetic modification, bioenergy supply through producing *α*-ketoacid, detoxification of ammonia, and maintaining intracellular redox status [61], which influence the subsequent hematology recovery postradiation. We noticed that the predicted pathways only suggested the potential interactions between host and gut microbiota, further studies were needed to reveal the mechanism by which gut microbes contributing to ARS. Taken together, we highlighted that the above-mentioned key microbes could serve as potential biomarkers to discriminate different phases of host with ARS.

In addition, we also found the well correlations between hematological indicators and bacterial species. Several well-known probiotics were identified in irradiated beagles such as *Lactobacillus acidophilus*, *Lactobacillus helveticus*, *Lactobacillus reuteri*, *Lactobacillus johnsonii*, *Lactobacillus gasseri*, *Lactobacillus lactis*, *Lactobacillus salivarius*, and *Lactobacillus animalis*. These microbes had been shown to significantly correlate with hematological factors [62–69]. For the decreased RBC and lymphocyte in postradiation, the negative correlations between hematological factors and probiotics were found, which were contradicted to the previous common sense that probiotics would decrease after irradiation. Moreover, the next-generation probiotics of *Bacteroides fragilis* and *Faecalibacterium prausnitzii* in irradiated beagles exhibited the opposite trends with previous reports [70, 71], which may afford potential immunomodulating functions. However, *Streptococcus mutans*, *Streptococcus gallolyticus*, *Streptococcus agalactiae*, *Streptococcus lutetiensis*, *Streptococcus suis* and *Enterococcus cecorum* are usually known as potential pathogenic species those may cause infections [72–77]. Yet, they were enriched at the recovery phase during ARS. From current reports and our research, it is clear that radiation not only causes changes in peripheral blood cells, but also alters the composition of gut microbiota, and there is a correlation between these two aspects. However, the mechanism of this correlation is currently not clear. On one hand, changes in gut microbiota led to changes in bacterial metabolites, which can enter tissues or blood through the intestinal barrier, leading to changes in the body, including changes in blood cells. On the other hand, changes in the composition and abundance of intestinal bacteria can lead to changes in some metabolic pathways, which in turn can cause changes in the body. In addition, the damage caused by radiation to the body may result in changes of certain molecules or cellular regulatory factors, which in turn affect gut microbiota. Therefore, under the key factor of radiation, there may also be mutual influence between peripheral blood cells and intestinal microbiota, but this still needs further verification.

Now, there is a rising interest in how the microbiome influences the individual's generally reaction to radiation and the possible mechanisms. The following aspects may help researchers gain a deeper understanding of the relationship and mechanism between gut microbiota and radiation. First is the screening for key gut bacteria and metabolites associated with radiation damage through analysis of changes in gut microbiota and their metabolites. Then, the increased or decreased abundance of these bacteria or metabolites are needed to uncover the potential mechanisms. In addition, what metabolic pathways are involved and how they are regulated by gut microbiota are also main focus.

## 5. Conclusions

Our study offers the relevant preclinical details on the dynamics of gut microbiota in dog postradiation. The characteristically altered gut microbes induced by irradiation greatly differ at the various clinical phases, which is likely to serve as biomarkers to discriminate the different phases of patients with ARS. At the latency phase *Lactobacillus*, *Ruminococcus*, and *Clostridioides* at genus level, at the manifest illness phase *Micrococcales* and *Peptostreptococcaceae at* family level, and at the recovery phase *Streptococcus and Enterococcus* at genus level should be focused on.

## Figures and Tables

**Table 1:** Changes of gut microbiota in different irradiated animal models.

Study objects	Methods	Changed species	Reference
Mice	16s rDNA	Enrichment: *Helicobacter, Alistipes* Decrease: *Lactobacillus*, *Ruminococcus*, and *Streptococcus*	[56]
Wistar rats	Quantitative PCR	Enrichment: *Bacteroidales, Lactobacillaceae,* and *Streptococcaceae* Decrease: *Clostridiaceae*	[57]
Beagles	Metagenomics	Enrichment: *Lactobacillus, Ruminococcus, Clostridioides, Micrococcales*, *Peptostreptococcaceae, Streptococcus,* and *Enterococcus* Decrease: *Bacteroides plebeius, Fusobacterium, Prevotella, Sutterella wadsworthensis*	This study

## Data Availability

Sequencing datasets have been deposited to the NCBI Sequence Read Archive under BioProject accession number PRJNA898082.

## Nomenclature

ARS: Acute radiation syndrome
Gy: Gray
PCA: Principal component analysis
LDA: Linear discriminant analysis
LEfSe: Linear discriminant analysis effect size
KEGG: Kyoto encyclopedia of genes and genomes
KOs: Kyoto encyclopedia of genes and genomes orthologys
tRNA: Transfer ribonucleic acid
ABC: ATP-binding cassette
RBC: Red blood cell
PLTs: Platelets
LYMPH: Lymphocytes
EO: Eosinophils
BASO: Basophils
HGB: Hemoglobin
HCT: Hematocrit
MPV: Mean platelet volume
MONO: Monocyte
DNA: Deoxyribonucleic acid
EDTA: Ethylenediaminetetraacetic acid
PCR: Polymerase chain reaction

## Supporting Information

Supporting Information 1 Supporting Figure 1 (Figure S1): Microbial co-occurrence network at different timepoints before and after radiation.

Supporting Information 2 Supporting Figure 2 (Figure S2): Relative abundance of each bacterial species with significantly changed after radiation at different timepoints.

Supporting Information 3 Supporting Table 1 (Table S1): Statistical values of hemotological data.

Supporting Information 4 Supporting Table 2 (Table S2): All hemotological data of Beagles at different timepoints before and after radiation.
