# Supplementary figures and images for: Alterations in Gut Microbiota Correlate With Hematological Injuries Induced by Radiation in Beagles
Source: Int J Microbiol. 2024 Dec 3;2024:3096783. doi: 10.1155/ijm/3096783 (PMC11631345; doi:10.1155/ijm/3096783)

# Co-occurrence network

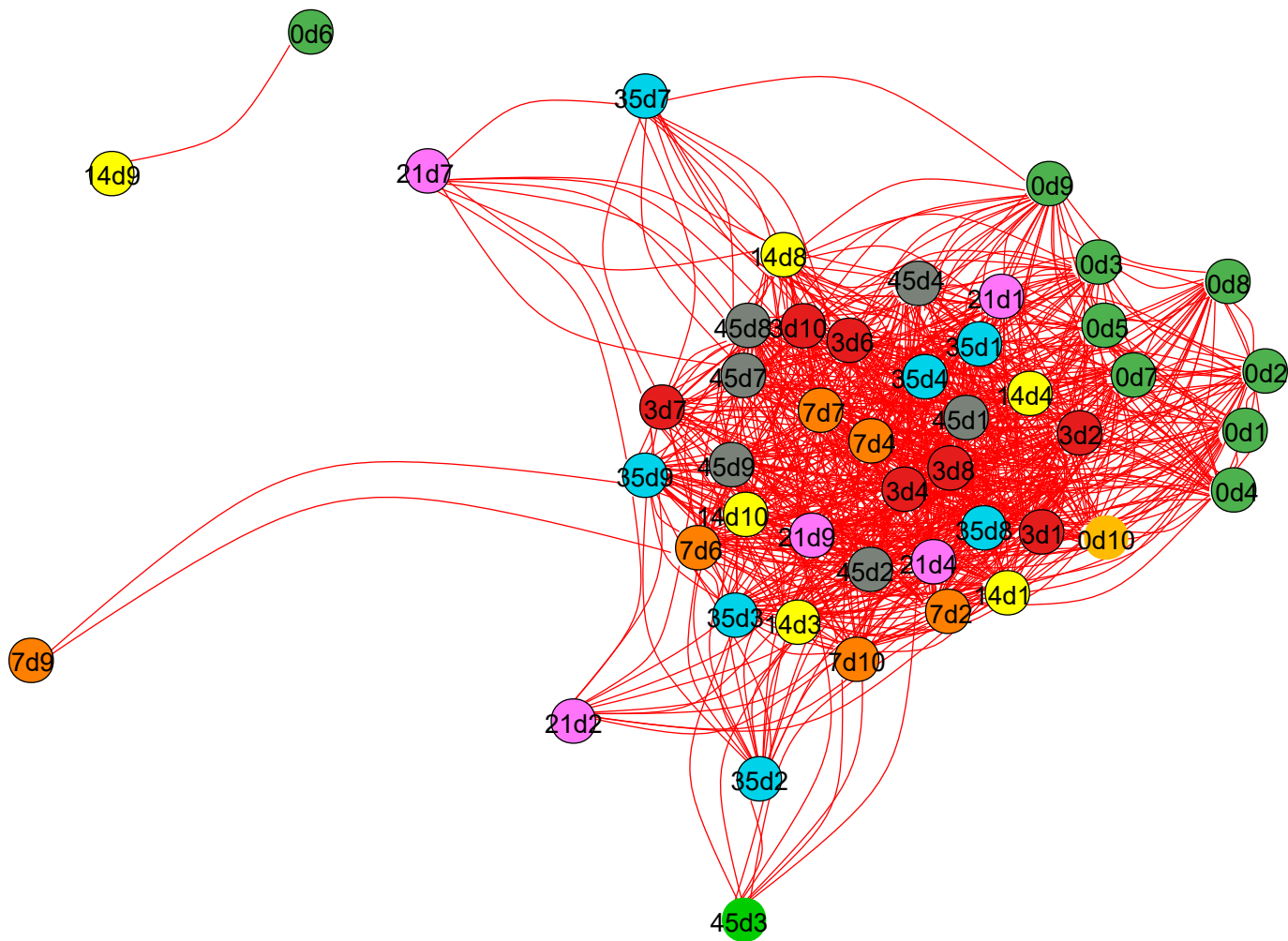

Supplement: Supporting Information 1 — Supporting Figure 1 (Figure S1): Microbial co-occurrence network at different timepoints before and after radiation. [file 3096783.f1.pdf]

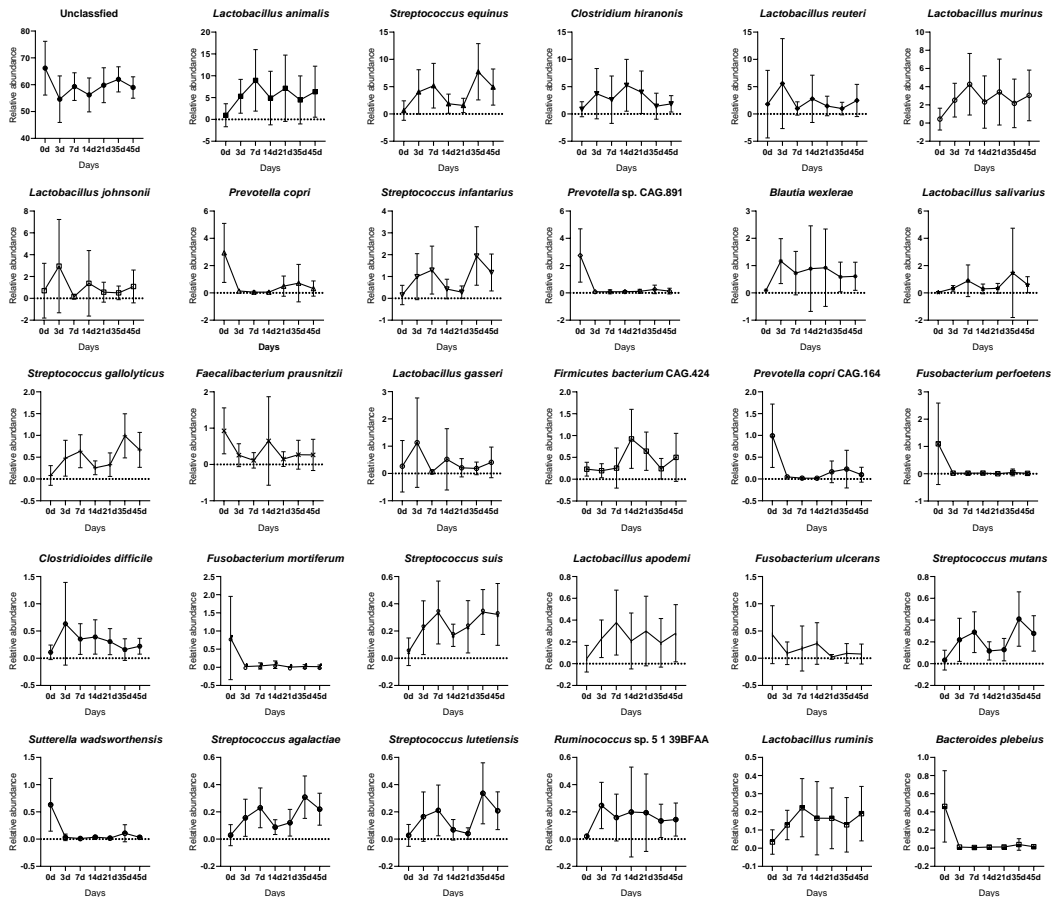

Supplement: Supporting Information 2 — Supporting Figure 2 (Figure S2): Relative abundance of each bacterial species with significantly changed after radiation at different timepoints. [file 3096783.f2.pdf]
