# Supplementary material for: Alterations in Gut Microbiota Correlate With Hematological Injuries Induced by Radiation in Beagles
Source: Int J Microbiol. 2024 Dec 3;2024:3096783. doi: 10.1155/ijm/3096783 (PMC11631345; doi:10.1155/ijm/3096783)
Supplement: Supporting Information 3 — Supporting Table 1 (Table S1): Statistical values of hemotological data. [file 3096783.f3.docx]

Table S1 Statistical values of hemotological data

|  | **WBC** | **NEUT** | **PLT** | **HCT** |
| --- | --- | --- | --- | --- |
| **0d** | 13.50±4.698 | 7.817±3.355 | 312.0±82.07 | 51.16±2.957 |
| **7d** | 3.128±0.7974 | 1.728±0.7441 | 123.4±57.15 | 47.61±3.513 |
| **vs 0d** | p<0.0001; F=34.71 | p<0.0001; F=20.32 | p<0.0001; F=3.062 | p=0.0250; F=1.411 |
| **14d** | 1.546±0.6963 | 0.5578±0.4599 | 0.7778±1.716 | 44.47±3.891 |
| **vs 0d** | p<0.0001; F=45.52 | p<0.0001; F=53.21 | p<0.0001; F=2288 | p=0.0005; F=1.731 |
| **21d** | 2.038±1.122 | 0.7863±0.5681 | 9.125±12.47 | 39.61±5.689 |
| **vs 0d** | p <0.0001; F=17.54 | p <0.0001; F=34.87 | p <0.0001; F=43.30 | p <0.0001; F=3.702 |
| **35d** | 9.849±3.570 | 6.564±2.337 | 194.9±100.0 | 45.90±3.780 |
| **vs 0d** | p= 0.0886; F=1.732 | p= 0.3842; F=2.061 | p= 0.0148; F=1.485 | p= 0.0044; F=1.634 |
| **45d** | 12.68±2.538 | 8.714±1.550 | 317.0±149.87 | 46.90±3.383 |
| **vs 0d** | p= 0.6630; F=3.425 | p= 0.7817; F=4.681 | p= 0.8819; F=2.709 | p= 0.0116; F=1.309 |
