# Supplementary material for: Alterations in Gut Microbiota Correlate With Hematological Injuries Induced by Radiation in Beagles
Source: Int J Microbiol. 2024 Dec 3;2024:3096783. doi: 10.1155/ijm/3096783 (PMC11631345; doi:10.1155/ijm/3096783)
Supplement: Supporting Information 4 — Supporting Table 2 (Table S2): All hemotological data of Beagles at different timepoints before and after radiation. [file 3096783.f4.pdf]

Seq.	Sample ID	Analysis	RBC(M/uL)	HGB(g/dL)	HCT(%)	MCV(fL)	MCH(pg)	MCHC(g/dI
0d	0d1	WB	7.16	15.3	44.6	62.3	21.4	34.3
0d	0d2	WB	8.02	18.9	55.9	69.7	23.6	33.8
0d	0d3	WB	7.77	17.3	51.2	65.9	22.3	33.8
0d	0d4	WB	8.26	18.1	53.8	65.1	21.9	33.6
0d	0d5	WB	8.45	18.6	56.7	67.1	22	32.8
0d	0d6	WB	7.89	17.9	51.7	65.5	22.7	34.6
0d	0d7	WB	8.64	17.9	52.2	60.4	20.7	34.3
0d	0d8	WB	7.6	16.7	49.8	65.5	22	33.5
0d	0d9	WB	7.99	17	51.3	64.2	21.3	33.1
0d	0d10	WB	7.81	17.8	52.6	67.3	22.8	33.8
7d	7d1	WB	7.82	17.8	52	66.5	22.8	34.2
7d	7d2	WB	8.17	16.8	51	62.4	20.6	32.9
7d	7d3	WB	6.85	14.8	44.5	65	21.6	33.3
7d	7d4	WB	6.96	16	47.1	67.7	23	34
7d	7d5	WB	6.35	14.7	43.3	68.2	23.1	33.9
7d	7d6	WB	7.49	16.5	49.5	66.1	22	33.3
7d	7d7	WB	7.25	15.6	46.1	63.6	21.5	33.8
7d	7d8	WB	6.42	14.4	42	65.4	22.4	34.3
7d	7d9	WB	7.99	17.4	50.4	63.1	21.8	34.5
7d	7d10	WB	7.91	16.8	50.2	63.5	21.2	33.5
14d	14d1	WB	7.45	17.3	48.4	65	23.2	35.7
14d	14d2	WB	7.26	15.4	44.6	61.4	21.2	34.5
14d	14d3	WB	6.32	13.9	39.7	62.8	22	35
14d	14d4	WB	6.72	15.7	44.7	66.5	23.4	35.1
14d	14d5	WB	6.31	14.3	40.6	64.3	22.7	35.2
14d	14d6	WB	7.12	16	45.9	64.5	22.5	34.9
14d	14d7	WB	6.12	13.9	39.6	64.7	22.7	35.1
14d	14d8	WB	7.42	16.4	45.9	61.9	22.1	35.7
14d	14d9	WB	7.98	17.5	50.8	63.7	21.9	34.4
21d	21d1	WB	6.46	13.6	39.4	61	21.1	34.5
21d	21d2	WB	4.85	10.6	30.3	62.5	21.9	35
21d	21d3	WB	5.61	13	37.1	66.1	23.2	35
21d	21d4	WB	5.58	12.6	35.5	63.6	22.6	35.5
21d	21d5	WB	6.61	14.6	42.5	64.3	22.1	34.4
21d	21d6	WB	5.77	13.6	38.3	66.4	23.6	35.5
21d	21d7	WB	7.44	16.1	46.5	62.5	21.6	34.6
21d	21d8	WB	7.38	16.1	47.3	64.1	21.8	34
35d	35d1	WB	7.21	15.1	47.5	65.9	20.9	31.8
35d	35d2	WB	6.4	13.6	44.2	69.1	21.3	30.8
35d	35d3	WB	6.41	14.9	45.5	71	23.2	32.7
35d	35d4	WB	6.2	13.6	42.4	68.4	21.9	32.1
35d	35d5	WB	7.14	15.9	48.4	67.8	22.3	32.9
35d	35d6	WB	5.96	13.6	41.3	69.3	22.8	32.9
35d	35d7	WB	7.13	15.2	44.7	62.7	21.3	34
35d	35d8	WB	7.88	17.3	53.2	67.5	22	32.5
45d	45d1	WB	7.34	15.6	48.1	65.5	21.3	32.4
45d	45d2	WB	6.3	13.4	42.3	67.1	21.3	31.7
45d	45d3	WB	6.99	16	49.2	70.4	22.9	32.5
45d	45d4	WB	6.67	14.5	45.4	68.1	21.7	31.9
45d	45d5	WB	7.31	16	47.9	65.5	21.9	33.4
45d	45d6	WB	6.26	14	42.4	67.7	22.4	33
45d	45d7	WB	7.53	16	47.7	63.3	21.2	33.5
45d	45d8	WB	8.01	18	52.2	65.2	22.5	34.5

RDW-SD(fl	RDW-CV(%)	RBC-I(M/u	MCV-I(fL)	RET#(K/ul	RET%(%)	IRF(%)	LFR(%)	MFR(%)	HFR(%)
35.4	19	7.16	62.3	103.8	1.45	35.5	64.5	22.6	12.9
41.1	19.6	8.02	69.7	214.1	2.67	47	53	30.7	16.3
39.8	20.1	7.77	65.9	134.4	1.73	43.3	56.7	19.1	24.2
36.7	19.6	8.26	65.1	113.2	1.37	38.7	61.3	23.7	15
39.4	20	8.45	67.1	156.3	1.85	40.8	59.2	31.7	9.1
36.4	18.3	7.89	65.5	65.5	0.83	19.3	80.7	12.2	7.1
38	21.6	8.64	60.4	70	0.81	21.4	78.6	15.1	6.3
39.8	20.2	7.6	65.5	161.9	2.13	33.4	66.6	23.5	9.9
39.5	20.7	7.99	64.2	169.4	2.12	30.2	69.8	23	7.2
42.6	20.8	7.81	67.3	208.5	2.67	39.3	60.7	25.8	13.5
35.3	18.3	7.82	66.5	4.7	0.06	0	100	0	0
37.6	20.2	8.17	62.4	5.7	0.07	0	100	0	0
37.4	18.2	6.85	65	4.8	0.07	11.2	88.8	5.6	5.6
38.8	17.6	6.96	67.7	9.7	0.14	6.1	93.9	0	6.1
36	15.8	6.35	68.2	4.4	0.07	37.5	62.5	12.5	25
38.1	19.5	7.49	66.1	7.5	0.1	16	84	4	12
38.5	19.6	7.25	63.6	18.1	0.25	9.8	90.2	4.9	4.9
34.6	17	6.42	65.4	10.3	0.16	15	85	5	10
37.8	19.9	7.99	63.1	13.6	0.17	0	100	0	0
35.6	19	7.91	63.5	5.5	0.07	5.9	94.1	5.9	0
33.6	16.4	7.45	65	11.9	0.16	35.9	64.1	20.5	15.4
36.1	19.4	7.26	61.4	5.8	0.08	40	60	25	15
35.1	17.5	6.32	62.8	7	0.11	33.3	66.7	11.1	22.2
36.6	16.6	6.72	66.5	4	0.06	35.7	64.3	14.3	21.4
36.1	17.3	6.31	64.3	6.3	0.1	24	76	20	4
37.7	19.9	7.12	64.5	49.1	0.69	38.9	61.1	22.4	16.5
33.8	16.5	6.12	64.7	11	0.18	25	75	18.2	6.8
35.6	18.5	7.42	61.9	0.7	0.01	0	100	0	0
35.8	20.1	7.98	63.7	89.4	1.12	39.1	60.9	26.4	12.7
35.5	18.6	6.46	61	7.8	0.12	30	70	6.7	23.3
33.6	15.8	4.85	62.5	28.1	0.58	20.3	79.7	6.3	14
37	15.8	5.61	66.1	11.8	0.21	29.4	70.6	15.7	13.7
35.4	17.6	5.58	63.6	13.4	0.24	45.7	54.3	22	23.7
37.7	20.4	6.61	64.3	95.8	1.45	47.5	52.5	23.9	23.6
33.5	18.8	5.77	66.4	35.8	0.62	43.1	56.9	23.5	19.6
35.8	19	7.44	62.5	11.2	0.15	26.3	73.7	18.4	7.9
38	21.1	7.38	64.1	98.2	1.33	29.3	70.7	22.9	6.4
46.6	23.5	7.21	65.9	212.7	2.95	32.8	67.2	24.2	8.6
49.1	22.6	6.4	69.1	278.4	4.35	32	68	25.3	6.7
46.8	21.7	6.41	71	222.4	3.47	36	64	27.5	8.5
44.9	22.6	6.2	68.4	256.7	4.14	39.3	60.7	30.3	9
45.6	22.3	7.14	67.8	237.8	3.33	55.8	44.2	30.9	24.9
42.1	21.4	5.96	69.3	168.1	2.82	40.2	59.8	28	12.2
40	21.4	7.13	62.7	199.6	2.8	35.7	64.3	25.6	10.1
45.5	22.2	7.88	67.5	154.4	1.96	27.5	72.5	22.8	4.7
45.3	22.2	7.34	65.5	168.1	2.29	25.9	74.1	17.8	8.1
43.8	20.8	6.3	67.1	239.4	3.8	34.5	65.5	25.3	9.2
45.5	20.9	6.99	70.4	288.7	4.13	46.8	53.2	30.1	16.7
46.3	21.9	6.67	68.1	312.2	4.68	38	62	26.9	11.1
41	20.8	7.31	65.5	200.3	2.74	49.7	50.3	24.4	25.3
42.2	20.9	6.26	67.7	172.8	2.76	43.6	56.4	28.9	14.7
40.9	21.7	7.53	63.3	218.4	2.9	36.5	63.5	26.9	9.6
40.7	21.3	8.01	65.2	212.3	2.65	48.6	51.4	31.2	17.4

RBC-O(M/u	PLT-O(K/u	MCV-O(fL)	MPV-O(fL)	RBC-X(ch)	RBC-Y(ch)	RBC-Z(ch)	PLT(K/uL)	PDW(fL)	MPV(fL)
7.72	322	72.2	12.8	12.7	138	143.8	373	14.5	11.3
8.66	332	76.7	13	12.9	144.9	145.6	398	15.5	11.9
8.31	250	74.6	12.1	14.5	141.9	143.9	274	15.7	12
9.25	84	73.4	12.4	14.2	140.2	143.8	106	15.9	11.9
8.99	278	73.4	12.7	12.4	140	143.6	309	14.3	11
8.52	260	75.1	12.6	13.5	142.7	146	283	14.5	11
9.25	297	69.6	12.9	13.2	134.3	142.8	328	15.2	11.2
8.21	289	74.3	13	11.4	141.4	145.4	343	15.9	11.2
8.53	337	72.6	13.4	12.5	138.9	144.9	363	15.1	11.2
8.46	324	77.7	13.7	13.6	146.3	147.7	343	16.2	11.8
8.62	22	75.9	14.4	13.6	143.8	146.2	17	----	----
8.72	134	71	14.1	13.6	136.5	144.3	148	----	----
7.31	152	72.6	13.6	13	139	145.1	168	17.7	12
7.57	95	77.9	13.5	14.7	146.8	148.8	97	15.6	11.7
6.9	79	76.7	14	13.9	145.1	148	69	18.5	13.2
8.22	149	74	13.2	12.2	141	147.4	149	15.6	11.4
7.78	169	73.8	12.8	16.9	140.7	150	181	13.7	10.1
7.02	87	72.8	13.2	15	139.1	152	73	13.5	10.8
8.7	147	72.4	14.5	14.9	138.5	150.5	137	18.2	11.8
7.96	162	72.9	12.3	13.6	139.1	147.2	195	14.3	10.6
8.2	0	74.6	11.7	12.5	141.9	146.1	0	----	11.7
8.13	0	69.1	13.9	11.9	133.7	143	0	----	----
6.99	0	70.7	13.9	11.2	135.9	144.5	0	----	----
7.39	0	75.5	15.3	12.4	143.2	146.6	0	----	----
7.04	0	71.7	14	10.8	137.6	145.5	0	----	14
7.9	5	72.2	16	14	138.3	144.8	5	----	16
7.01	0	72.9	13.7	11.5	139.1	147.6	0	----	13.7
8.23	0	70.6	12.8	12	135.8	149.2	0	----	12.8
8.66	2	69.7	14.6	12.9	134.6	147.2	2	----	14.6
7.24	0	68.6	16.4	8.4	132.9	145.1	0	----	16.4
5.42	32	69.6	17	9.5	134.3	145.6	32	----	17
6.25	0	74.6	20	10.6	141.7	149	0	----	----
6.4	10	70.6	15.4	9.7	136	149.7	4	----	----
7.16	37	72.2	13.9	15.6	138.2	148.3	25	----	----
6.73	6	72.5	15.2	13.6	138.8	148.9	3	----	----
7.85	0	71.3	13.9	11.8	136.9	146.5	0	----	----
10.9	9	71.5	15	11.5	137.3	145.2	9	----	15
7.86	195	70.4	15.2	13.7	135.6	142.4	189	----	----
6.86	377	70.4	15	13.1	135.6	141.5	403	22.1	13.5
7.08	105	75.1	15.8	13.5	142.8	145	106	20.3	13.7
6.93	131	70.7	14.5	11.2	135.8	146.2	117	----	----
7.65	98	71.5	13.9	15.9	137.2	148.2	99	16.8	11.1
6.79	251	72.6	13.4	14	138.8	148.1	242	15.6	12
7.52	172	70.9	14.8	13.4	136.6	144.9	177	----	----
9.14	237	69.1	12.4	11.6	133.9	135.5	226	18.2	12
8.15	258	70.6	14.2	12.6	135.8	142.4	272	19.5	12.9
6.86	355	71.4	15.6	13	137.2	142.1	375	----	----
7.64	284	76.7	13.8	13.8	145	145.2	301	18.6	12.2
7.2	266	73.2	13.7	12.3	139.8	144.9	278	18.1	11.9
7.9	241	73.3	13.6	15.3	139.7	148.8	288	17.4	11.9
6.87	311	73.4	13.5	13.4	139.8	149.4	336	16.4	11.2
8.36	284	70.1	14.4	13.2	135.1	146.5	281	22.4	12.8
11.01	460	72.2	11.6	13.7	138.1	145.9	405	13	10.2

P-LCR(%)	PCT(%)	PLT-I(K/u	MPV-I(fL)	WBC(K/uL)	NEUT#(K/u	LYMPH#(K/	MONO#(K/u	EO#(K/uL)	BASO#(K/u
37.2	0.42	373	11.3	18.81	11.72	5.55	1.25	0.27	0.02
41.2	0.47	398	11.9	13.34	7.98	3.56	1.01	0.72	0.07
40.3	0.33	274	12	22.83	14.64	6.74	1.15	0.26	0.04
41	0.13	106	11.9	11.86	6.77	4.24	0.64	0.2	0.01
34.5	0.34	309	11	11.85	7.39	3.44	0.7	0.27	0.05
34.1	0.31	283	11	6.39	2.63	2.17	1.22	0.31	0.06
36.4	0.37	328	11.2	10	5.1	3.7	0.84	0.26	0.1
36.8	0.38	343	11.2	10.14	5.96	3.26	0.78	0.13	0.01
36.9	0.41	363	11.2	15.06	8.43	4.46	1.53	0.64	0
40.7	0.4	343	11.8	14.69	7.55	5.31	1.32	0.5	0.01
----	----	17	----	1.83	0.99	0.45	0.27	0.12	0
----	----	148	----	4.27	3.28	0.65	0.23	0.11	0
41.7	0.2	168	12	2.7	1.2	1.01	0.29	0.2	0
40.1	0.11	97	11.7	4.01	2.45	0.96	0.24	0.36	0
52.3	0.09	69	13.2	2.89	0.72	1.41	0.61	0.15	0
39.1	0.17	149	11.4	2.47	1.53	0.65	0.16	0.12	0.01
27.9	0.18	181	10.1	3.87	2	1.23	0.43	0.21	0
33.4	0.08	73	10.8	2.81	1.52	0.69	0.35	0.24	0.01
44.1	0.16	137	11.8	2.66	1.64	0.68	0.18	0.16	0
32.3	0.21	195	10.6	3.77	1.95	1.07	0.29	0.46	0
----	0	1	----	0.8	0.12	0.55	0.12	0.01	0
----	----	0	----	1.1	0.39	0.57	0.13	0.01	0
----	----	0	----	1.24	0.25	0.77	0.21	0.01	0
----	----	0	----	1.27	0.17	0.93	0.16	0	0.01
----	0	0	----	1.83	1.03	0.66	0.14	0	0
----	0.01	7	----	2.46	0.84	1.39	0.2	0.02	0.01
----	0	0	----	1.45	0.57	0.67	0.19	0.01	0.01
----	0	0	----	0.93	0.2	0.61	0.12	0	0
----	0	8	----	2.83	1.45	1.2	0.16	0.02	0
----	0	0	----	1.52	0.43	0.8	0.29	0	0
----	0.05	20	----	3.24	1.47	1.24	0.53	0	0
----	----	0	----	1.28	0.37	0.7	0.21	0	0
----	----	4	----	1.09	0.31	0.59	0.19	0	0
----	----	25	----	4.21	1.81	1.87	0.51	0.02	0
----	----	3	----	1.84	0.62	0.98	0.23	0.01	0
----	----	0	----	1.11	0.37	0.59	0.15	0	0
----	0.01	7	----	2.01	0.91	0.94	0.16	0	0
----	----	189	----	7.26	4.91	1.5	0.83	0.02	0
53.2	0.54	403	13.5	15.18	9.75	2.34	2.87	0.2	0.02
54.9	0.14	106	13.7	11.31	7.54	2.07	1.62	0.08	0
----	----	117	----	9.11	6.16	1.67	1.22	0.03	0.03
37.8	0.11	99	11.1	13.3	8.31	3.36	1.56	0.06	0.01
40.3	0.29	242	12	11.5	8.54	1.64	1.25	0.05	0.02
----	----	177	----	5.72	4.03	1.12	0.54	0.03	0
42	0.27	226	12	5.41	3.27	1.57	0.46	0.1	0.01
52.3	0.35	272	12.9	10.06	6.9	1.86	1.09	0.17	0.04
----	----	375	----	15.9	10.72	2.48	2.21	0.48	0.01
45.8	0.37	301	12.2	15.14	9.57	3.36	1.74	0.46	0.01
42.5	0.33	278	11.9	10.25	7.08	2.05	1.02	0.08	0.02
41.6	0.34	288	11.9	13.55	8.91	2.88	1.4	0.34	0.02
37.9	0.38	336	11.2	14.94	10.58	2.3	1.7	0.26	0.1
49.5	0.36	281	12.8	9.59	7.11	1.53	0.83	0.11	0.01
28.4	0.41	405	10.2	11.97	8.84	2.17	0.65	0.3	0.01

NEUT%(%)	LYMPH%(%	MONO%(%)	EO%(%)	BASO%(%)	DIFF-X(ch	DIFF-Y(ch	FSC-X(ch)	RET-He(pg	RBC-He(pg
62.4	29.5	6.6	1.4	0.1	87.6	46.2	10.3	22.5	22.6
59.8	26.7	7.6	5.4	0.5	89	47.3	10.8	23.5	24.2
64.2	29.5	5	1.1	0.2	87	46.5	10.5	23.3	23.5
57	35.8	5.4	1.7	0.1	85.8	45.1	10.8	22.7	23.1
62.4	29	5.9	2.3	0.4	88.5	47	10.4	23.2	23
41.1	34	19.1	4.9	0.9	85.5	46.9	10.6	24.1	23.7
51	37	8.4	2.6	1	84.7	46.5	10.2	22	21.8
58.8	32.1	7.7	1.3	0.1	88.9	46.6	10.7	23.5	23.4
56	29.6	10.2	4.2	0	89.8	48.5	10.5	21.9	22.8
51.4	36.1	9	3.4	0.1	82.2	47.5	10.2	24.3	24.5
54	24.6	14.8	6.6	0	88.3	57.2	11.8	22.9	23.9
76.8	15.2	5.4	2.6	0	86.9	52.9	10.7	21	22.3
44.5	37.4	10.7	7.4	0	92.4	58.2	12.1	24.2	22.8
61.1	23.9	6	9	0	84.8	52.3	11.3	20.9	24.6
24.9	48.8	21.1	5.2	0	102.4	94	11.6	23.9	24.2
61.9	26.3	6.5	4.9	0.4	89.2	49.2	12.1	23.7	23.3
51.7	31.8	11.1	5.4	0	91.8	53.6	11.2	23.2	23.2
54	24.6	12.5	8.5	0.4	89.6	52.7	12.3	19.2	22.8
61.6	25.6	6.8	6	0	86.3	51.1	11.4	20.3	22.7
51.7	28.4	7.7	12.2	0	88.1	49.4	12.2	22.7	22.8
14.9	68.8	15	1.3	0	90.3	68.9	11	23.1	23.5
35.5	51.8	11.8	0.9	0	89.9	59.2	10.5	22.6	21.7
20.2	62.1	16.9	0.8	0	96.8	54	11.1	19.6	22.1
13.4	73.2	12.6	0	0.8	88.6	58.5	11	21.9	23.8
56.2	36.1	7.7	0	0	94.2	51.3	10.9	21.7	22.5
34.2	56.5	8.1	0.8	0.4	94.1	54.8	10.5	21.7	22.7
39.3	46.2	13.1	0.7	0.7	93.5	58.7	10.9	23.1	22.8
21.5	65.6	12.9	0	0	95.5	63	11	22	22.1
51.2	42.4	5.7	0.7	0	90.3	47	10.3	22.7	21.9
28.3	52.6	19.1	0	0	95.5	76.7	10.6	22.6	21.5
45.3	38.3	16.4	0	0	95.4	59.6	11.3	21.1	21.8
28.9	54.7	16.4	0	0	95.8	84.6	10.9	25.2	23.4
28.5	54.1	17.4	0	0	101.7	88.1	10.7	22.5	22.2
43	44.4	12.1	0.5	0	92.4	57.7	9.9	21.7	22.6
33.7	53.3	12.5	0.5	0	93.2	64.1	10.9	22.3	22.8
33.3	53.2	13.5	0	0	97	82	11	21.2	22.4
45.2	46.8	8	0	0	92.9	56.4	10.2	21.3	22.4
67.6	20.7	11.4	0.3	0	84.2	53.4	11.3	20.8	22.1
64.3	15.4	18.9	1.3	0.1	87.6	51.8	12.5	20.1	22.1
66.7	18.3	14.3	0.7	0	81.6	54	11	21.5	23.7
67.7	18.3	13.4	0.3	0.3	86.5	49.5	12.8	22.3	22.1
62.4	25.3	11.7	0.5	0.1	87.3	51.3	12.4	22.5	22.4
74.2	14.3	10.9	0.4	0.2	85.7	51.5	11.9	23.4	22.8
70.5	19.6	9.4	0.5	0	86.2	55.8	11.7	20.1	22.3
60.5	29	8.5	1.8	0.2	86.8	50.3	10.4	20.3	21.7
68.6	18.5	10.8	1.7	0.4	84.7	46.8	9.9	22	22.1
67.4	15.6	13.9	3	0.1	89.1	47.2	11.5	20.9	22.4
63.2	22.2	11.5	3	0.1	82.2	48.2	10.2	23.6	24.2
69	20	10	0.8	0.2	88.1	45.8	11.1	23.1	23
65.8	21.3	10.3	2.5	0.1	88	46.6	10.7	23.8	23
70.8	15.4	11.4	1.7	0.7	86.5	47.7	11.3	24.9	23
74.1	16	8.7	1.1	0.1	85.5	48.6	10.3	23	22
73.9	18.1	5.4	2.5	0.1	86.6	47.9	10.5	23	22.6

RET-Y(ch)	RBC Total	RBC Rate	PLT Total	PLT Rate	R-MFV	PDW-CV	S-RBC	S-MCV	S-RDW
137.7	9230	13	8719	7	642	10000	0	0	0
142.1	9384	14	8779	4	732	10000	0	0	0
141.3	9891	13	9752	5	684	10000	0	0	0
138.4	9673	14	8913	6	672	10000	0	0	0
140.9	9829	14	9159	5	703	10000	0	0	0
144.5	9294	14	9958	5	685	10000	0	0	0
135.3	9318	15	9338	8	624	10000	0	0	0
141.8	9698	13	8542	6	675	10000	0	0	0
134.6	9377	14	9678	6	660	10000	0	0	0
145.5	9914	13	8812	5	701	10000	0	0	0
139.3	9295	14	9342	5	690	0	0	0	0
130.6	9585	14	9279	7	648	0	0	0	0
144.9	9685	12	9911	5	670	10000	0	0	0
130	9834	12	9596	4	704	10000	0	0	0
143.6	9936	11	8455	4	707	10000	0	0	0
142.7	9635	13	9841	5	680	10000	0	0	0
140.9	9389	13	9358	6	658	10000	0	0	0
121.3	9209	12	9186	5	670	10000	0	0	0
127	9431	14	8690	7	656	10000	0	0	0
138.6	9360	14	8649	7	658	10000	0	0	0
140.4	9606	13	9871	5	671	0	0	0	0
137.8	9398	13	8885	7	629	0	0	0	0
123.7	9904	11	8751	6	639	0	0	0	0
135	9553	12	9634	4	691	0	0	0	0
133.9	9891	11	9333	5	657	0	0	0	0
133.8	9260	13	8431	6	659	0	0	0	0
140.2	9640	11	8963	5	660	0	0	0	0
135.3	9572	13	8651	7	639	0	0	0	0
138.4	9432	14	9718	6	651	0	0	0	0
138.2	9271	12	8659	7	620	0	0	0	0
131	9651	9	9258	5	629	0	0	0	0
149	9861	10	9129	4	684	0	0	0	0
137.4	9809	10	9489	5	641	0	0	0	0
134	9436	12	8745	6	646	0	0	0	0
136.6	9207	11	8775	5	656	0	0	0	0
131.7	9612	13	9965	6	647	0	0	0	0
131.8	9556	13	9517	6	644	0	0	0	0
129.5	9323	13	8630	6	658	0	0	0	0
126.3	9098	12	9934	4	694	10000	0	0	0
132.9	9197	12	7964	4	715	10000	0	0	0
136.7	9751	11	8210	5	673	0	0	0	0
137.5	9302	13	8721	5	687	10000	0	0	0
141.7	9420	11	9441	4	686	10000	0	0	0
126	9247	13	9958	6	635	0	0	0	0
127.2	9323	14	9360	5	692	10000	0	0	0
135.1	9418	13	9820	5	673	10000	0	0	0
130.2	9768	11	8043	5	688	0	0	0	0
142.3	9814	12	8165	4	727	10000	0	0	0
140.3	9448	12	8081	5	691	10000	0	0	0
143.3	9419	13	9974	5	671	10000	0	0	0
147.8	9768	11	8406	5	684	10000	0	0	0
139.7	9631	13	9855	6	649	10000	0	0	0
139.9	9426	14	9497	6	668	10000	0	0	0

P-MFV	PCT	L-RBC	L-MCV	L-RDW	PLT-I	RBC-LD	RBC-MD	RBC-UD	PLT-LD
99	42	0	0	0	373	0		49	0
105	47	0	0	0	398	0		49	0
99	33	0	0	0	274	0		49	1
101	13	0	0	0	106	0		49	1
94	34	0	0	0	309	0		49	0
94	31	0	0	0	283	0		49	0
99	37	0	0	0	328	0		49	0
95	38	0	0	0	343	0		49	0
96	41	0	0	0	363	0		49	0
103	40	0	0	0	343	0		49	0
84	0	0	0	0	17	0		49	2
115	0	0	0	0	148	0		49	1
93	20	0	0	0	168	0		49	0
99	11	0	0	0	97	0		49	1
111	9	0	0	0	69	0		49	2
92	17	0	0	0	149	0		49	1
83	18	0	0	0	181	0		49	0
86	8	0	0	0	73	0		49	0
101	16	0	0	0	137	0		49	1
87	21	0	0	0	195	0		49	1
145	0	0	0	0	1	0		49	0
0	0	0	0	0	0	0		49	0
284	0	0	0	0	0	0		49	0
85	0	0	0	0	0	0		49	5
60	0	0	0	0	0	0		49	4
75	1	0	0	0	7	0		49	4
291	0	0	0	0	0	0		49	0
80	0	0	0	0	0	0		49	0
130	0	0	0	0	8	0		49	0
65	0	0	0	0	0	0		49	4
133	5	0	0	0	20	0		49	1
55	0	0	0	0	0	0		49	3
108	0	0	0	0	4	0		49	2
89	0	0	0	0	25	0		49	2
69	0	0	0	0	3	0		49	2
289	0	0	0	0	0	0		49	0
91	1	0	0	0	7	0		49	3
124	0	0	0	0	189	0		49	2
113	54	0	0	0	403	0		49	0
108	14	0	0	0	106	0		49	1
94	0	0	0	0	117	0		49	0
92	11	0	0	0	99	0		49	0
89	29	0	0	0	242	0		49	0
111	0	0	0	0	177	0		49	0
91	27	0	0	0	226	0		49	0
119	35	0	0	0	272	0		49	1
115	0	0	0	0	375	0		49	0
107	37	0	0	0	301	0		49	0
95	33	0	0	0	278	0		49	0
96	34	0	0	0	288	0		49	0
95	38	0	0	0	336	0		49	0
109	36	0	0	0	281	0		49	1
85	41	0	0	0	405	0		49	0

PLT-UD	HGB Sampl	HGB Blank	RBC Clog	RBC Distr	PLT Distr	DIFF Tota	DIFF Rate	DIFF-X	DIFF-Y
28	4652	3123	98			9095	2	876	462
28	5006	3117	98			6582	2	890	473
27	4838	3111	98			7301	3	870	465
25	4918	3106	98			5539	2	858	451
26	4959	3099	98			5852	2	885	470
28	4879	3092	98			6337	1	855	469
24	4868	3082	98			5046	2	847	465
26	4737	3069	98			9950	1	889	466
26	4756	3059	98			7756	2	898	485
28	4836	3052	97			7269	2	822	475
28	4464	2679	97		UD DW	2082	1	883	572
26	4346	2670	97		DW	4202	1	869	529
28	4136	2660	97			3113	1	924	582
27	4249	2650	97			3893	1	848	523
27	4108	2642	97			2830	1	1024	940
25	4283	2632	96			2435	1	892	492
22	4188	2626	96			3907	1	918	536
26	4066	2621	96			2724	1	896	527
22	4356	2617	95			2825	1	863	511
25	4297	2616	95			3749	1	881	494
28	4683	2956	97		UD DW	773	1	903	689
24	4486	2949	97		DW	1075	1	899	592
27	4329	2941	97		DW	1201	1	968	540
27	4507	2934	97		DW	1189	1	886	585
28	4354	2927	97		UD DW	1731	1	942	513
29	4521	2923	97		UD DW	2376	1	941	548
28	4310	2916	96		UD DW	1360	1	935	587
26	4550	2910	96		UD DW	901	1	955	630
29	4653	2904	96		UD DW	2917	1	903	470
25	3941	2581	96		UD DW	1588	1	955	767
24	3628	2570	96		UD DW	4616	1	954	596
26	3865	2562	96		DW	1282	1	958	846
20	3810	2550	95		DW	1245	1	1017	881
22	4009	2545	95		DW	5105	1	924	577
27	3901	2542	94		DW	1824	1	932	641
25	4154	2540	94		DW	1114	1	970	820
25	4144	2539	94		UD DW	2025	1	929	564
27	4104	2594	96		DW	8974	1	842	534
28	3945	2581	96			9344	2	876	518
28	4057	2569	96			5862	2	816	540
26	3921	2559	96		DW	9211	1	865	495
23	4133	2546	95			7214	2	873	513
29	3896	2536	95			5737	2	857	515
25	4055	2532	94		DW	6899	1	862	558
26	4260	2528	94			5999	1	868	503
24	4444	2888	98			5610	2	847	468
28	4211	2875	98		DW	6938	3	891	472
25	4461	2861	97			7755	2	822	482
27	4296	2847	97			5280	2	881	458
27	4430	2833	97			7056	2	880	466
25	4216	2819	97			7364	2	865	477
26	4404	2804	96			5343	2	855	486
26	4582	2786	96			6166	2	866	479

DIFF-WX	DIFF-WY	NEUT-X	NEUT-Y	LYMPH-X	LYMPH-Y	MONO-X	MONO-Y	NEUT-WX	NEUT-WY
508	1729	876	462	829	882	1068	1553	343	585
488	629	890	473	855	983	1069	1595	326	613
474	623	870	465	847	954	1064	1509	333	603
467	596	858	451	854	992	1045	1592	338	576
469	573	885	470	845	962	1059	1547	328	554
580	657	855	469	874	1024	1063	1733	351	555
531	578	847	465	848	1020	1063	1659	366	559
490	535	889	466	849	968	1062	1686	326	493
473	593	898	485	853	971	1055	1566	334	577
566	626	822	475	847	1001	1056	1518	365	632
914	2336	883	572	886	1094	1057	1570	340	682
379	604	869	529	881	1095	1041	1534	322	605
450	1492	924	582	867	1037	1030	1502	325	601
375	569	848	523	867	1071	1101	1533	342	573
883	1087	1024	940	851	1075	1108	1245	381	564
417	545	892	492	863	977	1053	1476	325	528
539	560	918	536	867	1029	1059	1570	327	560
513	584	896	527	889	1125	1064	1563	313	607
415	486	863	511	877	1110	1041	1548	325	470
469	556	881	494	856	1001	1024	1514	318	486
468	1165	903	689	917	1082	1018	1383	277	580
398	1282	899	592	902	1067	995	1425	334	794
606	956	968	540	899	991	1133	1407	362	944
400	970	886	585	898	1041	1032	1455	327	666
345	584	942	513	903	997	1013	1312	276	584
396	1937	941	548	891	999	1046	1327	340	693
463	1964	935	587	914	1112	1005	1523	278	715
390	3890	955	630	915	1090	1082	1403	335	778
378	552	903	470	897	1005	1063	1508	310	553
621	2072	955	767	920	1076	1081	1475	377	717
489	2189	954	596	903	1068	1071	1549	325	637
566	917	958	846	896	1121	1049	1550	355	697
897	2653	1017	881	903	1049	1011	1582	384	624
823	10417	924	577	885	1015	1072	1726	346	641
457	1626	932	641	902	1152	1012	1588	290	562
512	8767	970	820	904	1059	1044	1475	320	731
440	1877	929	564	874	1015	992	1421	312	586
577	543	842	534	877	1117	1059	1575	344	505
533	540	876	518	907	1037	1058	1733	342	541
607	537	816	540	873	1124	1060	1715	356	463
528	484	865	495	897	1022	1069	1751	358	464
549	1637	873	513	873	932	1049	1734	355	604
522	524	857	515	897	1130	1054	1746	350	505
810	574	862	558	878	1090	1043	1876	348	502
544	476	868	503	847	1060	1028	1621	346	437
563	512	847	468	882	1022	1062	1705	342	513
471	1750	891	472	906	1035	1060	1644	326	593
591	600	822	482	880	1049	1058	1555	365	560
477	523	881	458	880	992	1067	1674	340	502
465	556	880	466	880	1008	1044	1657	329	537
495	502	865	477	903	1078	1070	1704	312	482
478	493	855	486	893	1053	1046	1706	316	493
439	500	866	479	865	1050	1040	1624	323	481

LYMPH-WX	LYMPH-WY	MONO-WX	MONO-WY	WBC#(Dif	WBC#(FSC)	Delta-WB(	WBC-LD	FSC-X	FSCW-Inf
471	816	290	728	18808	18016	1044	0	103	0
421	723	271	765	13343	13037	1023	0	108	1
449	755	282	630	22826	21694	1052	0	105	0
363	766	297	691	11859	10972	1081	0	108	2
438	748	255	718	11854	11581	1024	0	104	0
377	771	320	641	6387	6270	1019	0	106	0
401	696	320	778	9995	9986	1001	0	102	1
400	826	320	658	10135	9838	1030	0	107	1
410	782	265	658	15063	15339	982	0	105	0
402	709	256	791	14692	14366	1023	0	102	0
395	777	473	465	1832	2037	899	0	118	68
329	749	423	378	4271	4110	1039	0	107	0
392	762	417	686	2696	2459	1096	7	121	1
381	719	418	248	4011	3805	1054	0	113	3
482	670	415	554	2887	2766	1044	0	116	3
406	880	361	237	2472	2377	1040	0	121	0
392	719	331	834	3871	3815	1015	0	112	0
315	676	507	525	2814	2658	1059	0	123	2
342	676	413	174	2659	2757	965	0	114	2
421	779	449	238	3774	3658	1032	0	122	0
338	721	413	72	798	761	1048	0	110	0
299	721	402	84	1101	1059	1040	0	105	0
389	686	406	142	1243	1182	1051	0	111	0
312	663	397	110	1268	1170	1084	0	110	0
310	772	365	107	1834	1704	1077	0	109	0
303	751	306	384	2455	2337	1050	0	105	0
295	684	458	256	1447	1337	1082	0	109	0
284	670	360	150	928	886	1047	0	110	0
312	736	348	192	2834	2867	988	0	103	1
359	660	453	129	1521	1547	983	0	106	0
388	740	336	884	3242	3018	1074	7	113	3
324	544	438	194	1282	1248	1027	0	109	0
432	791	455	190	1091	1212	900	0	107	2
384	886	298	811	4214	4971	848	0	99	0
344	651	425	504	1837	1774	1035	0	109	0
343	718	278	102	1106	1084	1021	0	110	0
320	680	363	267	2009	1968	1021	0	102	0
376	761	293	762	7260	6735	1078	7	113	0
386	829	255	675	15179	14095	1077	7	125	1
401	774	274	700	11309	11423	990	0	110	1
424	822	318	600	9108	8299	1097	7	128	0
504	923	305	738	13303	12323	1079	7	124	1
390	726	323	779	11504	11165	1030	0	119	0
421	771	336	603	5720	5306	1078	7	117	0
413	839	282	796	5405	5837	926	0	104	0
374	782	264	739	10055	11032	911	0	99	0
397	811	236	736	15903	14919	1066	7	115	2
409	801	265	740	15140	15240	993	0	102	0
477	826	272	825	10254	10366	989	0	111	1
466	823	326	724	13550	13853	978	0	107	3
366	779	252	716	14937	14448	1034	0	113	1
381	845	268	627	9594	10482	915	0	103	0
393	771	279	727	11974	12089	990	0	105	0

FSCW-Inf	DIFF LD I	RET Total	RET Rate	RBC-X	RBC-Y	RBC-Z	RET-X	RET-Y	RBC-WX
2	5185	84703	1	127	1380	1438	639	1377	709
0	5185	92652	1	129	1449	1456	712	1421	700
1	5185	88837	1	145	1419	1439	691	1413	689
0	5185	94574	1	142	1402	1438	649	1384	705
0	5185	94774	1	124	1400	1436	668	1409	723
0	5185	90528	1	135	1427	1460	562	1445	665
0	5185	97054	1	132	1343	1428	577	1353	760
0	5124	88328	1	114	1414	1454	620	1418	793
0	5124	91648	1	125	1389	1449	643	1346	718
0	5124	90934	1	136	1463	1477	665	1455	659
179	4941	88123	1	136	1438	1462	375	1393	660
1	4941	90276	1	136	1365	1443	386	1306	661
0	4941	78541	1	130	1390	1451	451	1449	771
3	4941	80037	1	147	1468	1488	358	1300	610
0	4941	73936	1	139	1451	1480	517	1436	717
1	4941	86186	1	122	1410	1474	419	1427	822
0	4941	82747	1	169	1407	1500	455	1409	651
0	4941	75032	1	150	1391	1520	415	1213	667
2	4941	90189	1	149	1385	1505	382	1270	671
0	4941	84227	1	136	1391	1472	466	1386	659
0	5063	84507	1	125	1419	1461	649	1404	717
1	5063	83888	1	119	1337	1430	657	1378	759
0	5063	73906	1	112	1359	1445	600	1237	803
0	5063	77376	1	124	1432	1466	621	1350	725
2	5063	74383	1	108	1376	1455	642	1339	833
0	5063	81946	1	140	1383	1448	679	1338	715
0	5063	74026	1	115	1391	1476	599	1402	786
0	5063	84740	1	120	1358	1492	323	1353	752
0	5063	88352	1	129	1346	1472	666	1384	697
0	4880	75831	1	84	1329	1451	558	1382	948
0	4880	59489	1	95	1343	1456	510	1310	945
0	4880	66812	1	106	1417	1490	632	1490	851
0	4880	68306	1	97	1360	1497	701	1374	927
0	4880	75621	1	156	1382	1483	702	1340	706
2	4880	71266	1	136	1388	1489	680	1366	663
1	4880	81121	1	118	1369	1465	563	1317	761
0	4880	105931	1	115	1373	1452	639	1318	783
0	4880	83761	1	137	1356	1424	655	1295	800
1	4880	77459	1	131	1356	1415	653	1263	838
2	4880	75865	1	135	1428	1450	664	1329	817
0	4880	74836	1	112	1358	1462	679	1367	889
0	4880	80732	1	159	1372	1482	750	1375	690
0	4880	75113	1	140	1388	1481	695	1417	715
0	4880	80508	1	134	1366	1449	688	1260	748
0	4880	94783	1	116	1339	1355	619	1272	776
0	5002	87280	1	126	1358	1424	611	1351	793
0	5002	77425	1	130	1372	1421	656	1302	770
2	5002	83341	1	138	1450	1452	720	1423	796
0	5002	79171	1	123	1398	1449	674	1403	810
1	5002	84989	1	153	1397	1488	734	1433	719
0	5002	76822	1	134	1398	1494	704	1478	745
0	5002	89229	1	132	1351	1465	681	1397	755
1	4941	112147	1	137	1381	1459	738	1399	729

RBC-WY	RBC-O	Delta-RB(	PLT-O	Delta-PLT	PLT Blank	THR	THR-H	UPP	W-Area
297	772	1078	322	863	0	39	15	0	153
297	866	1080	332	834	0	38	17	0	101
310	831	1069	250	912	0	40	21	0	160
293	925	1120	84	792	0	40	15	0	93
307	899	1064	278	900	0	38	20	0	129
287	852	1080	260	919	0	39	17	0	51
365	925	1071	297	905	0	42	5	0	79
311	821	1080	289	843	0	37	25	0	57
324	853	1068	337	928	0	39	24	0	143
301	846	1083	324	945	0	38	26	0	155
278	862	1102	22	1294	0	38	11	0	22
337	872	1067	134	905	0	41	8	0	31
317	731	1067	152	905	0	39	3	0	31
279	757	1088	95	979	0	39	5	0	36
283	690	1087	79	1145	0	39	4	0	15
305	822	1097	149	1000	0	38	7	0	6
299	778	1073	169	934	0	42	6	0	49
252	702	1093	87	1192	0	40	3	0	30
282	870	1089	147	1073	0	40	7	0	18
288	796	1006	162	831	0	40	5	0	29
289	820	1101	0	0	0	38	5	0	0
344	813	1120	0	0	0	40	5	0	8
331	699	1106	0	0	0	39	4	0	9
293	739	1100	0	0	0	38	3	0	13
320	704	1116	0	0	0	38	1	0	11
318	790	1110	5	714	0	41	12	0	8
280	701	1145	0	0	0	38	7	0	20
302	823	1109	0	0	0	39	9	0	15
297	866	1085	2	250	0	40	18	0	20
324	724	1121	0	0	0	38	1	0	7
313	542	1118	32	1600	0	37	12	0	25
282	625	1114	0	0	0	36	5	0	8
294	640	1147	10	2500	0	37	8	0	5
304	716	1083	37	1480	0	42	13	0	28
274	673	1166	6	2000	0	40	10	0	14
299	785	1055	0	0	0	38	2	0	14
299	1090	1477	9	1286	0	39	20	0	8
347	786	1090	195	1032	0	42	26	0	54
347	686	1072	377	935	0	41	47	0	123
301	708	1105	105	991	0	38	36	0	97
317	693	1118	131	1120	0	38	28	0	74
306	765	1071	98	990	0	42	33	0	100
288	679	1139	251	1037	0	41	23	0	93
315	752	1055	172	972	0	40	13	0	58
351	914	1160	237	1049	0	40	17	0	49
354	815	1110	258	949	0	41	23	0	80
335	686	1089	355	947	0	41	18	0	122
310	764	1093	284	944	0	39	37	0	115
336	720	1079	266	957	0	39	29	0	80
308	790	1081	241	837	0	42	25	0	103
293	687	1097	311	926	0	40	26	0	104
311	836	1110	284	1011	0	41	24	0	73
311	1101	1375	460	1136	0	42	15	0	86

RBC Flag	WBC Flg	RET LD Di	SFL	Counter	Monthly I	Piercer	Air Pump	Sheath Mo	Tmp_REACT
16	0	5612	103	57	20	33	57	244	408
8	0	5612	103	56	19	32	56	240	408
5	0	5612	103	55	18	31	55	236	408
9	0	5612	103	54	17	30	54	232	408
0	0	5612	103	53	16	29	53	228	408
6	0	5612	103	52	15	28	52	224	408
6	0	5612	103	51	14	27	51	220	408
13	0	5551	103	50	13	26	50	216	408
6	0	5551	103	49	12	25	49	212	408
3	3	5551	103	48	11	24	48	208	408
59	0	5368	103	72	33	46	72	306	408
8	0	5307	103	71	32	45	71	302	408
0	0	5368	103	70	31	44	70	298	408
10	0	5307	103	69	30	43	69	294	405
7	0	5307	103	68	29	42	68	290	408
8	0	5307	103	67	28	41	67	286	408
3	0	5307	103	66	27	40	66	282	408
5	0	5307	103	65	26	39	65	278	408
8	3	5307	103	64	25	38	64	274	408
10	0	5307	103	63	24	37	63	270	408
0	0	5490	103	87	45	58	87	369	408
8	0	5490	103	86	44	57	86	365	408
7	0	5490	103	85	43	56	85	361	408
12	0	5490	103	84	42	55	84	357	408
0	0	5490	103	83	41	54	83	353	408
3	0	5490	103	82	40	53	82	349	408
7	0	5490	103	81	39	52	81	345	408
8	0	5490	103	80	38	51	80	341	408
8	0	5490	103	79	37	50	79	337	408
9	0	5307	103	96	53	66	96	406	408
9	0	5307	103	95	52	65	95	402	408
4	0	5246	103	94	51	64	94	398	408
13	0	5246	103	93	50	63	93	394	408
16	0	5246	103	92	49	62	92	390	408
4	0	5246	103	91	48	61	91	386	408
5	0	5246	103	90	47	60	90	382	408
0	0	5246	103	89	46	59	89	378	405
15	0	5307	103	108	9	75	108	459	408
9	0	5246	103	107	8	74	107	455	405
12	0	5246	103	106	7	73	106	451	408
14	0	5246	103	105	6	72	105	447	408
10	0	5246	103	104	5	71	104	443	408
2	0	5246	103	103	4	70	103	439	408
7	0	5246	103	102	3	69	102	435	408
9	0	5246	103	101	2	68	101	431	405
5	3	5429	103	118	17	83	118	501	408
9	0	5429	103	117	16	82	117	497	408
20	0	5429	103	116	15	81	116	493	408
29	0	5368	103	115	14	80	115	489	405
16	0	5368	103	114	13	79	114	485	408
7	0	5368	103	113	12	78	113	481	408
8	0	5368	103	112	11	77	112	477	405
14	0	5368	103	111	10	76	111	473	405

Tmp_REAG	Tmp_REAG	Tmp_FCM_S	Tmp_ENV	PRS07-1	PRS07-2	PRS07-3	VAC240-1	VAC240-2	VAC240-3
408	408	248	259	553	0	0	327	0	0
408	408	248	259	563	0	0	327	0	0
408	403	248	259	553	0	0	327	0	0
408	408	248	259	563	0	0	327	0	0
410	408	245	256	563	0	0	327	0	0
408	408	245	256	553	0	0	327	0	0
408	412	245	256	553	0	0	327	0	0
410	408	245	254	563	0	0	327	0	0
408	410	245	254	563	0	0	327	0	0
410	408	245	252	563	0	0	327	0	0
417	417	212	222	553	0	0	327	0	0
417	421	209	220	563	0	0	327	0	0
417	419	209	220	563	0	0	327	0	0
421	417	209	218	572	0	0	327	0	0
421	419	209	218	572	0	0	327	0	0
421	421	209	215	563	0	0	327	0	0
424	421	209	215	572	0	0	327	0	0
419	419	209	213	572	0	0	327	0	0
421	421	209	213	563	0	0	322	0	0
417	421	212	213	563	0	0	322	0	0
412	412	232	243	553	0	0	327	0	0
412	410	232	243	553	0	0	327	0	0
412	410	232	240	553	0	0	327	0	0
410	410	230	240	553	0	0	327	0	0
410	412	230	240	553	0	0	327	0	0
414	410	230	238	553	0	0	327	0	0
414	412	230	238	553	0	0	327	0	0
414	412	230	238	553	0	0	322	0	0
414	414	232	236	563	0	0	322	0	0
424	419	207	208	572	0	0	327	0	0
421	424	205	208	563	0	0	327	0	0
421	424	205	206	563	0	0	327	0	0
421	424	205	206	563	0	0	327	0	0
421	424	205	206	572	0	0	327	0	0
421	421	205	204	582	0	0	327	0	0
419	424	205	204	563	0	0	327	0	0
424	426	207	201	563	0	0	327	0	0
417	419	203	211	572	0	0	327	0	0
417	417	203	211	563	0	0	327	0	0
419	421	203	208	563	0	0	327	0	0
419	421	200	208	572	0	0	327	0	0
419	419	200	206	563	0	0	327	0	0
419	421	200	204	582	0	0	327	0	0
421	424	203	204	563	0	0	322	0	0
421	424	203	204	563	0	0	327	0	0
412	414	225	236	553	0	0	327	0	0
412	414	225	236	563	0	0	327	0	0
412	414	225	234	553	0	0	322	0	0
417	412	225	231	553	0	0	322	0	0
417	419	223	231	572	0	0	327	0	0
417	417	223	229	563	0	0	327	0	0
417	419	223	229	563	0	0	327	0	0
417	419	223	227	563	0	0	327	0	0

Laser Ti	Aspiratio	Aspiratio	Aspiratio	LyMoBound	NeEoBound	LyGsBound	LyNeBound	NeMoBound	GsNeBound
1835	3212	6995	8735	15	0	0	62	0	0
1797	3199	7044	9271	37	0	0	4	0	0
1759	3195	7099	9456	50	0	0	20	0	1
1720	3199	5590	8907	23	0	0	17	0	1
1682	3167	7115	9481	18	0	0	9	0	2
1644	3174	7066	9465	13	1	0	0	0	0
1606	3155	7062	9567	15	0	0	0	0	1
1567	3146	7056	9157	26	0	0	6	0	3
1529	3138	7376	9469	53	0	0	25	0	2
1491	3127	7115	9586	41	1	0	20	0	2
2409	2737	6901	8893	4	4	0	6	0	0
2370	2714	6695	8413	0	0	0	3	0	0
2332	2708	6766	7973	9	0	0	10	0	0
2294	2717	7375	8382	2	0	0	1	0	0
2256	2696	6167	7716	15	0	0	43	10	0
2217	2689	6686	8289	3	0	0	0	0	0
2179	2682	6858	8185	7	0	0	0	0	0
2141	2683	6684	8043	3	0	0	0	0	0
2102	2684	8052	9288	1	0	0	0	0	0
2064	2649	8176	9097	2	0	0	1	0	0
2981	3039	7656	9220	3	0	0	1	0	0
2943	3040	7136	8573	9	0	0	2	0	0
2905	3022	6859	7758	3	0	0	3	0	0
2867	3034	7109	8420	3	0	0	2	0	0
2828	3020	7013	8038	5	0	0	0	0	0
2790	3004	7413	8688	8	0	0	3	0	0
2752	3004	6752	8078	7	0	0	3	0	0
2713	3005	7443	8884	4	0	0	2	0	0
2675	2965	8306	9411	4	0	0	0	0	0
3326	2640	6020	7321	10	0	0	15	0	0
3288	2630	5443	6426	16	0	0	7	0	0
3250	2613	6099	7468	1	0	0	15	0	0
3211	2620	5930	6957	1	0	0	7	1	0
3173	2608	6430	8065	21	0	0	10	0	0
3135	2606	6129	7514	4	0	0	1	0	0
3096	2629	6672	8299	1	0	0	15	1	0
3058	2586	7035	8961	5	0	0	1	0	0
3784	2677	6486	8181	26	0	0	17	0	2
3746	2672	5764	7405	40	0	0	46	0	3
3708	2650	5823	7908	45	0	0	65	0	3
3669	2641	5860	7653	30	0	0	6	0	0
3631	2639	6234	7990	35	2	0	72	0	2
3593	2626	6135	7634	23	0	0	18	0	1
3554	2638	6425	8003	13	0	0	24	0	0
3516	2593	7043	9104	13	0	0	11	0	0
4166	3020	6639	8838	17	0	0	6	0	3
4128	3009	5900	7878	52	0	0	28	0	0
4090	2995	7041	9091	52	0	0	50	0	3
4051	2984	6374	8190	32	1	0	26	0	0
4013	2956	6970	9010	38	0	0	21	0	1
3975	2949	6424	8334	32	0	0	4	0	1
3936	2936	7605	9356	11	1	0	16	0	1
3898	2882	7670	9248	16	0	0	9	0	5

MoEoBound	GsEoBound	GsBaBound	NeBaBound	LyBaBound	EoBaBoundaryCount
0	0	0	6	5	0
0	0	0	9	13	0
0	0	0	5	30	0
0	0	0	1	2	0
0	0	0	7	10	0
0	0	0	6	4	0
0	0	0	8	13	0
0	0	0	1	1	0
0	0	0	0	1	0
0	0	0	1	12	0
0	1	0	0	0	0
0	0	0	0	0	0
0	0	0	0	0	0
0	0	0	0	0	0
0	0	0	0	0	0
0	0	0	0	8	0
0	0	0	0	0	0
0	0	0	1	1	0
0	0	0	0	0	0
0	0	0	1	0	0
0	0	0	0	0	0
0	0	0	0	0	0
0	0	0	0	0	0
0	0	0	0	1	0
0	0	0	0	0	0
0	0	0	2	1	0
0	0	0	0	2	0
0	0	0	0	0	0
0	0	0	0	0	0
0	0	0	0	0	0
0	0	0	0	0	0
0	0	0	0	0	0
0	0	0	0	0	0
0	0	0	0	0	0
0	0	0	0	0	0
0	0	0	0	0	0
0	0	0	0	0	0
0	0	0	0	1	0
0	0	0	2	2	0
0	0	0	0	0	0
0	0	0	4	10	0
0	0	0	0	3	0
0	0	0	6	7	0
0	0	0	0	0	0
0	0	0	1	0	0
0	0	0	4	12	0
0	0	0	4	17	0
0	0	0	0	1	0
0	0	0	2	4	0
0	0	0	2	7	0
0	0	0	19	36	0
0	0	0	1	1	0
0	0	0	1	1	0
